# Supplementary material for: Facial expressions elicit multiplexed perceptions of emotion categories and dimensions
Source: Curr Biol. 2022 Jan 10;32(1):200–209.e6. doi: 10.1016/j.cub.2021.10.035 (PMC8751635; doi:10.1016/j.cub.2021.10.035)
Supplement: Document S1. Figures S1–S3 [file mmc1.pdf]

Current Biology, Volume *32*

Supplemental Information

Facial expressions elicit multiplexed perceptions of emotion categories and dimensions

Meng Liu, Yaocong Duan, Robin A.A. Ince, Chaona Chen, Oliver G.B. Garrod, Philippe G. Schyns, and Rachael E. Jack

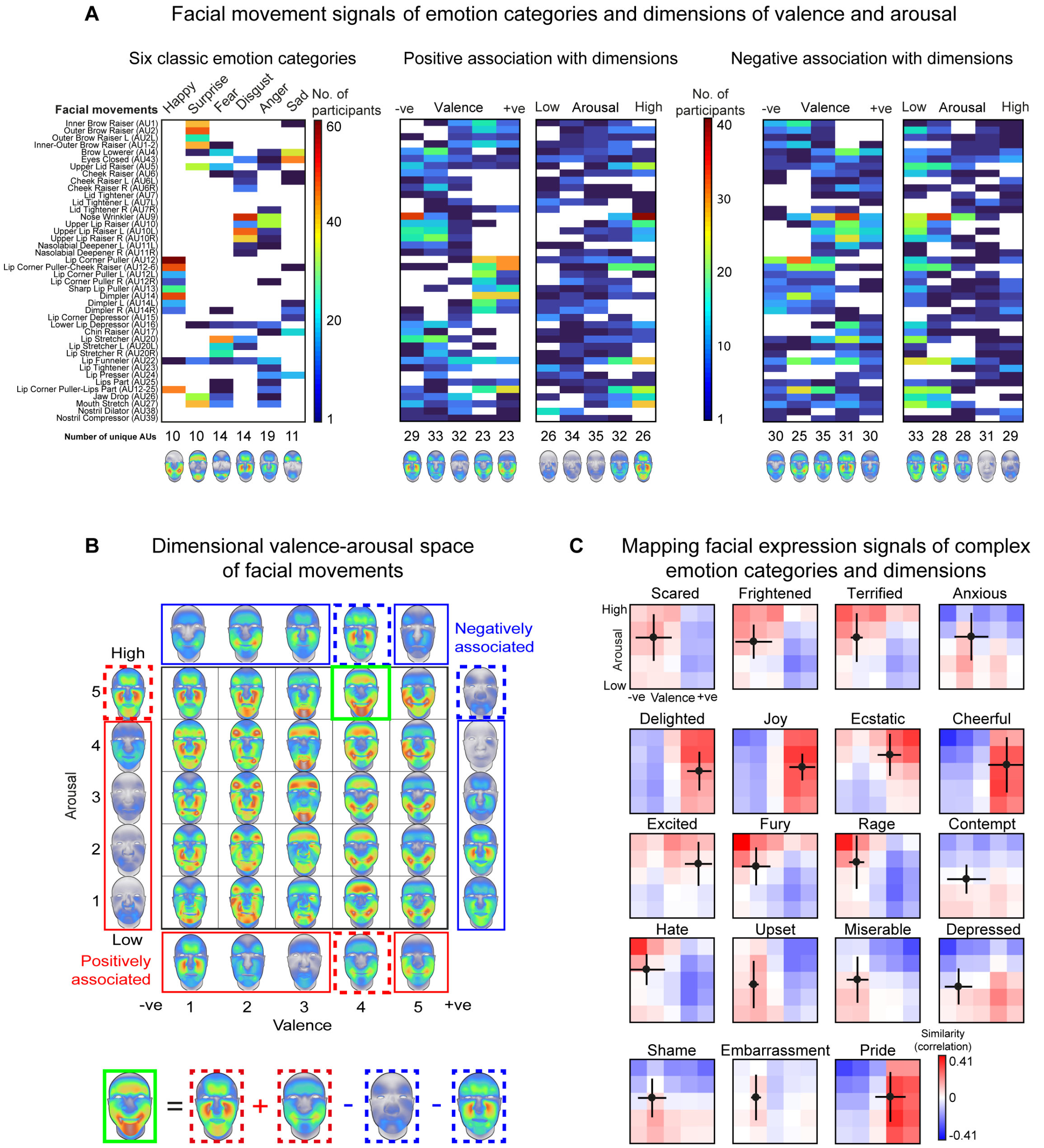

Figure S1. Modelling facial expression signals of emotion categories, and of emotion dimensions plus their mapping, Related to Figure 2, Experiment I in STAR Methods. (A) Facial movement signals of emotion categories and dimensional information. Each color-coded matrix shows the individual facial movements (i.e., Action Units) that signal emotion categories (left) or dimensional information (centre, right) as determined using Mutual Information (MI)[S1]. Color-coding refers to the number of participants (see colorbars). Warmer colors indicate higher numbers, cooler colors indicate lower numbers – for example, in emotion categories, Lip Corner Puller (AU12) is associated with ‘happy’ in all 60 participants (represented in dark red). For dimensional information, each matrix shows the AUs that are positively associated with perceptions of valence and arousal (centre) and negatively associated (right). For example, Nose Wrinkler (AU9) is positively associated with negative valence and negatively associated (i.e., disassociated) with positive valence and/or low arousal amongst most participants (represented in dark red – see color bar in centre). The total number of unique AUs and corresponding face

maps are shown below each emotion category and each dimensional rating. (B) Dimensional valence-arousal space of facial movements. Face maps in each cell of the 5 x 5 matrix show the facial movements associated with each combined level of valence and arousal, summed across participants. Each facial expression (see example outlined in green) is a combination of the corresponding positively associated individual Action Units (see dashed red outline) with any negatively associated AUs (dashed blue outline) removed. An example is shown below. (C) Mapping facial expression signals of complex emotions onto dimensions. Each color-coded sub-plot shows the average correlation between the facial expressions of a complex emotion (e.g., ‘ecstatic’) and the facial expressions of the dimensional valence-arousal space. Red indicates positive correlations; blue indicates negative correlations (see color bar on bottom right). For example, the facial expression of ‘ecstatic’ is positively correlated with facial expressions of high arousal positive valence, and negatively correlated with low arousal negative facial expressions. The semantic location of each emotion word is shown as a black point with crosses representing the standard deviation for each dimension[S2].

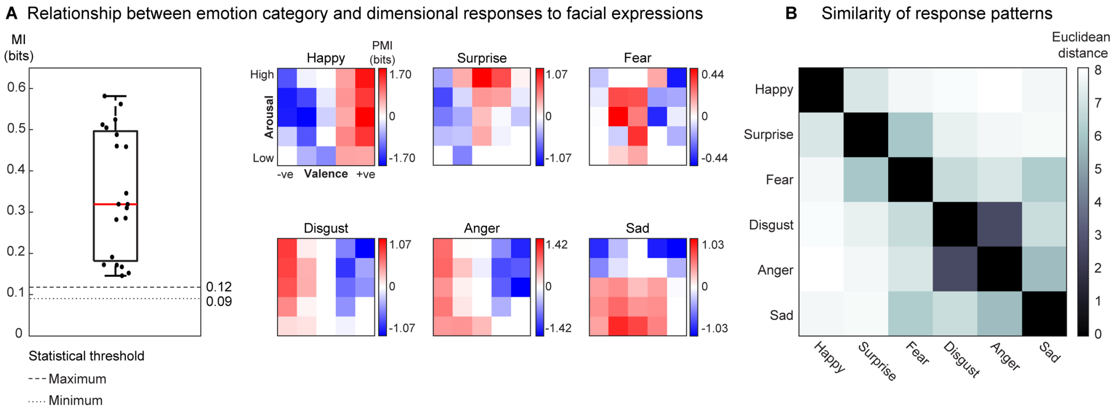

Figure S2. Relationship between emotion category responses and dimensional responses to facial expressions, Related to Experiment II, Replicating the link between the perception of emotion categories and dimensions in STAR Methods. (A) Relationship between emotion category and dimensional responses to facial expressions. The boxplot shows the Mutual Information (MI)[S1] values computed between the participants’ emotion category responses and their dimensional responses to the same facial expressions. Black points represent individual participants; the red line indicates the median; dashed lines show the minimum and maximum thresholds for statistical significance across participants. Color-coded matrices to the right show the specific relationship between the participants’ emotion category responses and their dimensional responses, measured using Pointwise Mutual Information (PMI)[S3]. Red indicates positive relationships; blue indicates negative relationships (see color bar to right) – for example, facial expressions categorized as ‘happy’ are often rated as ‘positive valence’ with varying levels of arousal. (B) Similarity of response patterns. To formally test the distinctiveness of these category-dimensional response relationships, we measured the pairwise similarities between the six distribution patterns. Results are shown as a gray-scale matrix. Darker squares indicate higher similarities; lighter squares indicate lower similarities (see colorbar to right). Results confirm that each emotion category is associated with a distinct range of valence and arousal ratings with overlap between disgust and anger, as commonly reported[S4–6].

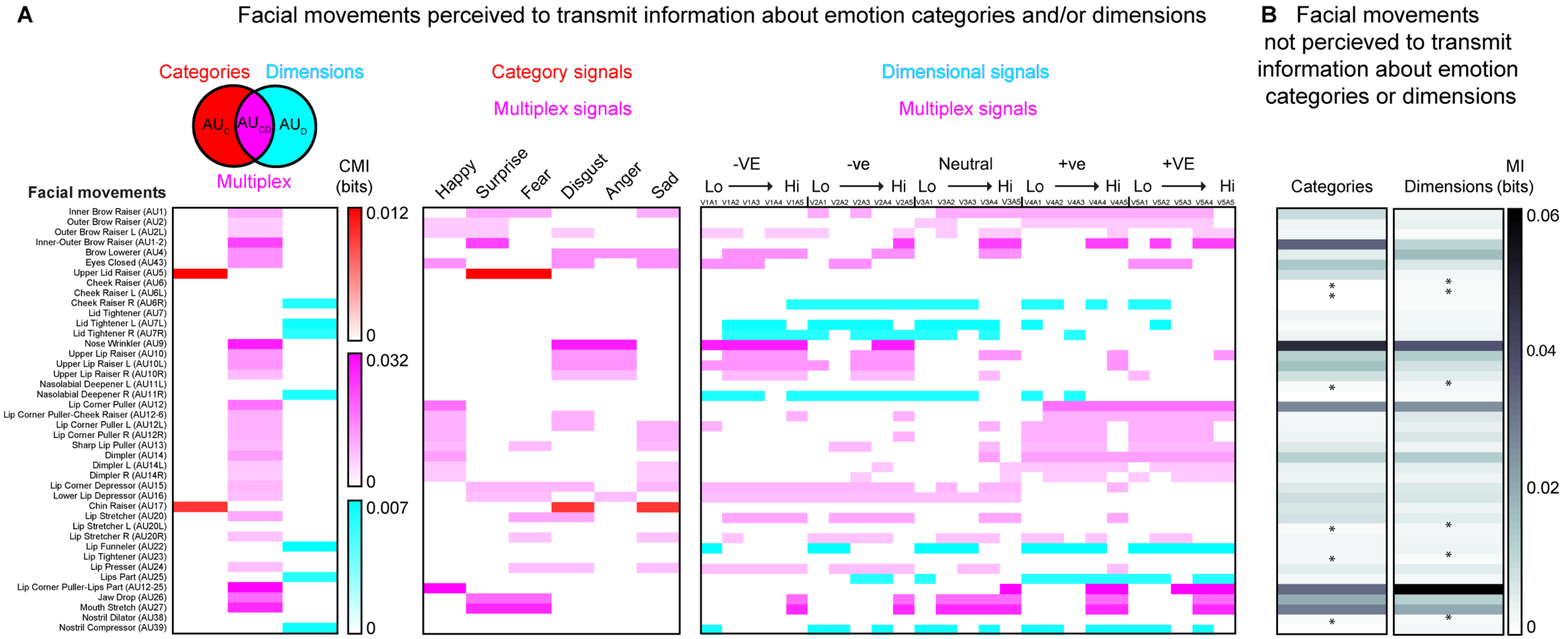

Figure S3. Facial movement signals that transmit information about emotion categories and/or dimensions, Related to Figure 3, Experiment II, Conditional Mutual Information analysis in STAR Methods. (A) Facial movement signals of emotion category and dimensional information. Left panel – each AU (see labels on y axis) is color-coded according to the categorical and/or dimensional information it transmits (see Venn diagram), derived using Conditional Mutual Information. Color saturation represents the CMI value, averaged across participants (see color bars to right). Centre and right panels – each color-coded matrix shows the specific emotion categories (represented in red, magenta) and dimensional information (represented in cyan, magenta) transmitted by each AU, derived using Pointwise Mutual Information (PMI). For example, Upper Lid Raiser (AU5) is an emotion category signal (left panel, red) that transmits information about the emotion categories 'fear' and 'surprise' (centre panel, red); the unilateral Lid Tighteners (AU7L/R) are dimensional signals (left panel, cyan) that transmit information about 'negative valence' across varying levels of arousal (right panel, cyan); Nose Wrinkler (AU9) is a multiplex signal (left panel, magenta) that transmits information about the emotion categories 'disgust' and 'anger' (centre panel, magenta) and the dimensions of 'negative valence' across varying levels of arousal (right panel, magenta). (B) Facial movements that do not transmit information about emotion categories or dimensions. Action Units identified by the CMI analysis as not influencing participant responses are indicated by asterisks. Gray-scale coding shows the Mutual Information (MI) between each AU and the participants' responses for the emotion categorization task (left column) and dimension rating task

(right column; see color bar on right). All AUs marked with an asterisk have low MI values in both tasks, confirming that they do not influence the participants’ responses.

SUPPLEMENTAL REFERENCES

S1. Ince, R.A.A., Giordano, B.L., Kayser, C., Rousselet, G.A., Gross, J., and Schyns, P.G. (2017). A statistical framework for neuroimaging data analysis based on mutual information estimated via a gaussian copula. Hum. Brain Mapp. *38*, 1541–1573.

S2. Warriner, A.B., Kuperman, V., and Brysbaert, M. (2013). Norms of valence, arousal, and dominance for 13,915 English lemmas. Behav. Res. Methods *45*, 1191–1207.

S3. Bouma, G. (2009). Normalized (pointwise) mutual information in collocation extraction. Proc. GSCL, 31–40.

S4. Jack, R.E., Garrod, O.G.B., and Schyns, P.G. (2014). Dynamic Facial Expressions of Emotion Transmit an Evolving Hierarchy of Signals over Time. Curr. Biol. *24*, 187–192.

S5. Aviezer, H., Hassin, R.R., Ryan, J., Grady, C., Susskind, J., Anderson, A., Moscovitch, M., and Bentin, S. (2008). Angry, disgusted, or afraid? Studies on the malleability of emotion perception. Psychol. Sci. *19*, 724–732.

S6. Widen, S.C., Russell, J.A., and Brooks, A. (2004). Anger and disgust: Discrete or overlapping categories. In 2004 APS Annual Convention, Boston College, Chicago, IL.
