# Supplementary material for: Facial expressions elicit multiplexed perceptions of emotion categories and dimensions
Source: Curr Biol. 2022 Jan 10;32(1):200–209.e6. doi: 10.1016/j.cub.2021.10.035 (PMC8751635; doi:10.1016/j.cub.2021.10.035)
Supplement: Document S2. Article plus supplemental information [file mmc3.pdf]

Current Biology

Report

Facial expressions elicit multiplexed perceptions of emotion categories and dimensions

Graphical Abstract

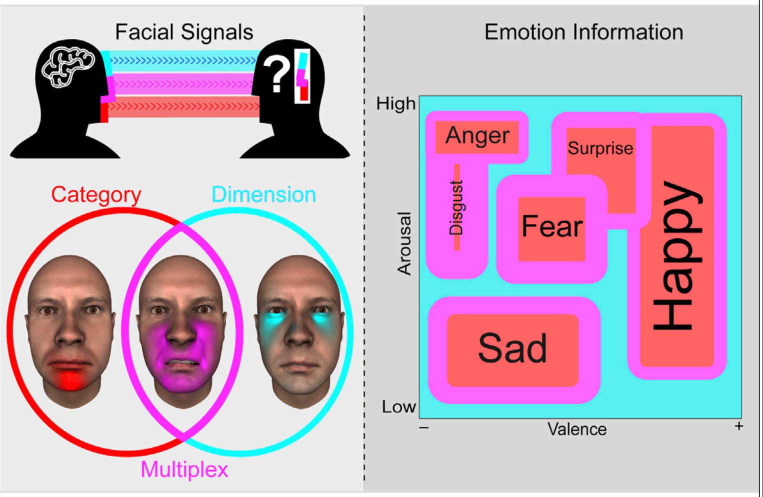

Authors

Meng Liu, Yaocong Duan, Robin A.A. Ince, Chaona Chen, Oliver G.B. Garrod, Philippe G. Schyns, Rachael E. Jack

Correspondence

rachael.jack@glasgow.ac.uk

In brief

Liu et al. examine how facial expressions signal broad-plus-specific emotion category and dimensional information. Using a perception-based facial-signal-modeling technique and information-theoretic analyses, they find a latent set of facial signals that can multiplex categorical and dimensional information and a subset uniquely signaling either.

Highlights

- Examined facial signals of broad-plus-specific emotion categories and dimensions
- Used data-driven, perception-based modeling and information-theoretic analyses
- Disentangled facial signals that multiplex broad-plus-specific emotion information
- Provides insights into facial expression ontology and new methodological framework

Liu et al., 2022, Current Biology *32*, 200–209
January 10, 2022 Crown Copyright © 2021 Published by Elsevier Inc.
https://doi.org/10.1016/j.cub.2021.10.035

CellPress

CellPress
OPEN ACCESS

Current Biology

Report

Facial expressions elicit multiplexed perceptions of emotion categories and dimensions

Meng Liu,[1] Yaocong Duan,[1] Robin A.A. Ince,[1] Chaona Chen,[1] Oliver G.B. Garrod,[1] Philippe G. Schyns,[1] and Rachael E. Jack[1,2,3,*]
[1]School of Psychology & Neuroscience, University of Glasgow, 62 Hillhead Street, Glasgow G12 8QB, UK
[2]Twitter: @rachaelejack
[3]Lead contact
*Correspondence: rachael.jack@glasgow.ac.uk
https://doi.org/10.1016/j.cub.2021.10.035

SUMMARY

Human facial expressions are complex, multi-component signals that can communicate rich information about emotions,[1–5] including specific categories, such as "anger," and broader dimensions, such as "negative valence, high arousal."[6–8] An enduring question is how this complex signaling is achieved. Communication theory predicts that multi-component signals could transmit each type of emotion information—i.e., specific categories and broader dimensions—via the same or different facial signal components, with implications for elucidating the system and ontology of facial expression communication.[9] We addressed this question using a communication-systems-based method that agnostically generates facial expressions and uses the receiver's perceptions to model the specific facial signal components that represent emotion category and dimensional information to them.[10–12] First, we derived the facial expressions that elicit the perception of emotion categories (i.e., the six classic emotions[13] plus 19 complex emotions[3]) and dimensions (i.e., valence and arousal) separately, in 60 individual participants. Comparison of these facial signals showed that they share subsets of components, suggesting that specific latent signals jointly represent—i.e., multiplex—categorical and dimensional information. Further examination revealed these specific latent signals and the joint information they represent. Our results—based on white Western participants, same-ethnicity face stimuli, and commonly used English emotion terms—show that facial expressions can jointly represent specific emotion categories and broad dimensions to perceivers via multiplexed facial signal components. Our results provide insights into the ontology and system of facial expression communication and a new information-theoretic framework that can characterize its complexities.

RESULTS AND DISCUSSION

Human facial expressions are complex dynamic signals composed of combinations of individual facial movements called action units (AUs)[14,15]—for example, smiles often comprise lip corner puller (AU12) and cheek raiser (AU6) and scowls often comprise brow lowerer (AU4), lid tightener (AU7), and upper lip raiser (AU10).[16] Current accounts report that facial expressions can provide complex combinations of specific emotion-category information and broader dimensional information[6,7] that could aid adaptive response.[13,17,18] Yet how facial expressions achieve this complex signaling task remains unknown because, while emotion category perceptions often predict (i.e., correlate with) dimensional perceptions of facial expressions, the specific facial signals that drive (i.e., explain) these perceptions are unknown. Communication theory predicts that such multi-component facial signals could transmit different types of information via the same components (e.g., lip corner puller, AU12) or different components (e.g., lip-corner puller, AU12 and cheek raiser, AU6), with specific implications for understanding the ontology and system of facial expression communication.[9] Here, we tested this hypothesis using a data-driven, perception-based methodology[19–27] to model and investigate three types of facial signals: those perceived to (1) specifically transmit emotion category information; (2) specifically transmit dimensional information; or (3) jointly transmit—i.e., multiplex—emotion category and dimensional information. Figure 1A schematizes these facial signals within a general framework of communication (see Shannon,[28] Bradbury and Vehrencamp,[29] Dukas,[30] Slater et al.,[31] and Scott-Phillips[32]).

Thus, understanding any system of communication—i.e., how information is transferred between individuals[28,29,30–32]—fundamentally relies on explaining what specific signals drive perceptual responses in receivers (see Jack and Schyns,[34] Schyns et al.,[35] Barrett et al.,[33] Krakauer et al.,[36] Wu et al.,[37] Naselaris et al.,[38] and Kriegeskorte and Douglas[39] for reviews and discussion). We examined this critical link between facial movements and their impact on receiver perception—here, of emotion categories, dimensions, or both—by combining classic data-driven reverse-correlation methods from ethology,[19] vision science,[20,21] neuroscience,[22–24] and engineering[25–27] (see Jack and Schyns[34] for a review) with a modern computer-graphics-based generative model of human facial movements,[10]

 Crown Copyright © 2021 Published by Elsevier Inc.
This is an open access article under the CC BY license (http://creativecommons.org/licenses/by/4.0/).

CellPress
OPEN ACCESS

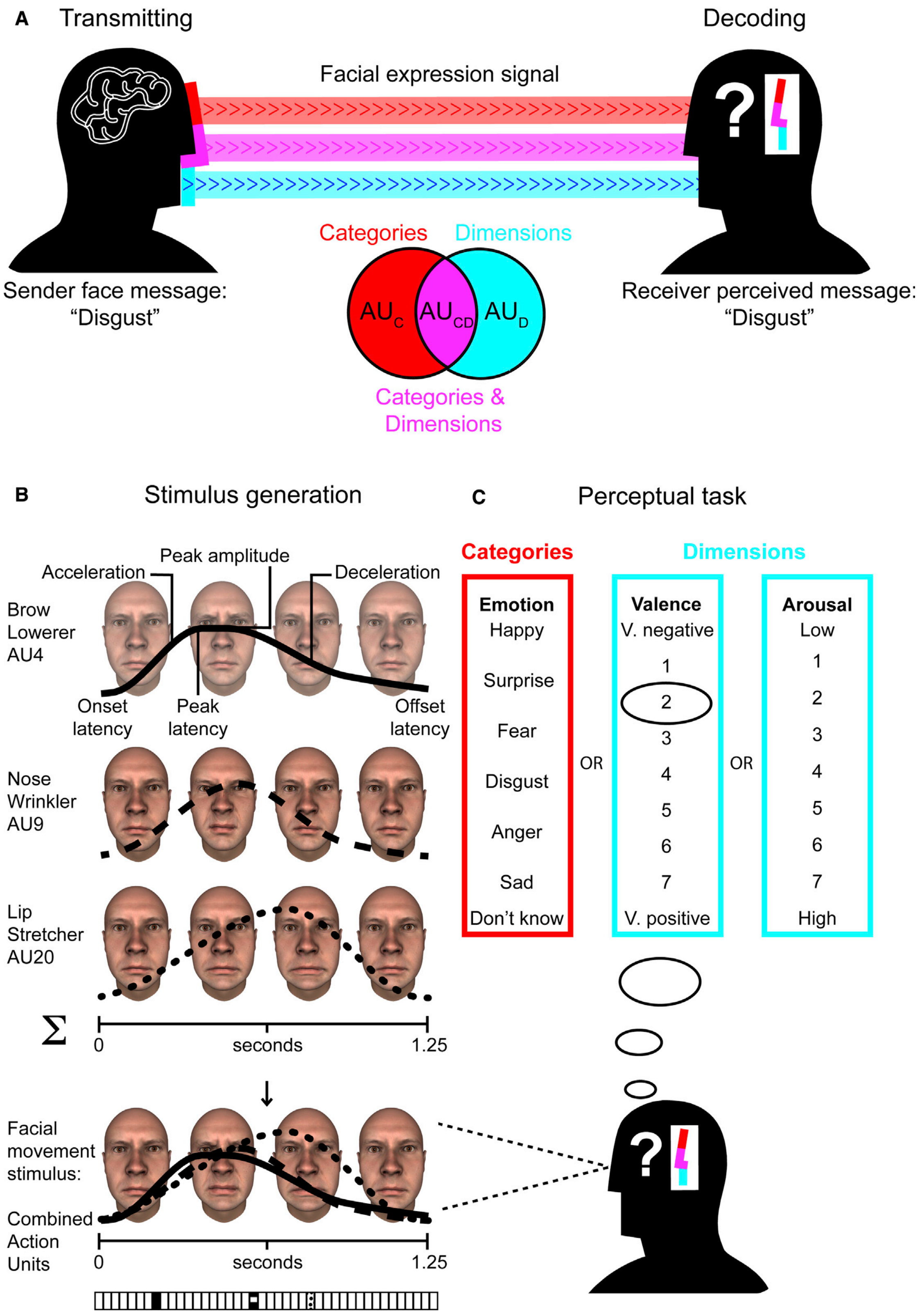

Figure 1. Multiplexed facial expressions of emotion: A new ontology

(A) Transmitting and decoding facial expression signals—illustration of the general system of communication[28,29] (see also Barrett et al.[33] for discussion). To communicate an emotion message to others—e.g., "disgust"—the sending face must encode the message into a perceptible signal, such as a facial expression.

(legend continued on next page)

subjective human perception,[3,40,41] and information-theoretic analysis tools.[11,12] Each step is described below.

Experiment I: Modeling facial expression signals of emotion categories and of dimensions

To understand which specific facial movements drive the perception of emotion categories and dimensions, we used the classic data-driven method of reverse correlation to agnostically generate facial expressions—i.e., random combinations of individual AUs—and then used the receiver's perceptual responses to isolate the specific facial movements that elicit their perception of emotion categories and/or dimensions. Figures 1B and 1C illustrate the procedure. On each experimental trial, a generative model of human facial movements[10] produced a facial expression stimulus by randomly selecting a combination of AUs and assigning a random movement to each AU (Figure 1B, labeled solid black curve; see Yu et al.;[10] Stimuli, Experiment I–STAR Methods). Figure 1B, bottom row, shows an example (see also Video S1). Each participant (100 white Western, English-speaking, 51 females, 49 males; Participants, Experiment I—STAR Methods) viewed the stimulus and interpreted it according to one of two pre-assigned tasks in a between-subjects design: (1) categorized as one of the six classic emotions—i.e., "happy," "surprise," "fear," "disgust," "anger," or "sad"—only if they perceived that the facial expression accurately represented that emotion category or "don't know" if they did not (Figure 1C, red frame) or (2) rated according to the dimensions of valence and arousal in separate counterbalanced blocks (Figure 1C, cyan frames). In Figure 1C, the participant perceived the randomly generated facial expression as transmitting "negative valence" (Figure 1C, black ellipse). Each experimental trial where the participant selected an emotion label thus captured a combination of dynamic AUs that elicited the participant's perception—e.g., "happy"—or dimensional message—e.g., "positive valence"—thus providing an estimate of their prior knowledge of these facial expressions, derived from their subjective experiences of the external world.[19,42,43] Each participant completed 2,400 trials, resulting in a large set of facial expressions associated with each response option (see Figures 2A and 2B bar plots for average across participants; Experiment I, Perceptual task procedure—STAR Methods).

Next, to isolate the specific facial movements that systematically elicit the perception of each emotion category and, separately, of each dimensional message, we measured the statistical relationship between the AUs presented on each trial and each participant's responses using a non-parametric measure of statistical dependence—mutual information (MI) (Experiment I, Facial expression modeling procedure—STAR Methods).[11] This produced, for each participant, a statistically robust model of the facial movements that elicit the perception of each emotion category—i.e., "happy," "surprise," "fear," "disgust," "anger," and "sad"—and each dimensional message—i.e., "very negative valence" to "very positive valence" and "very high arousal" to "very low arousal," represented as a two-dimensional valence-arousal space (Experiment 1, Facial expression modeling procedure—STAR Methods; Figures S1A and S1B illustrate the procedure). This resulted in a total of 360 facial expression models of the six classic emotion categories (60 participants × 6 emotion categories) and 1,000 facial expression models of dimensional messages (40 participants × 25 valence-arousal combinations across the 5 × 5 valence-arousal space). Figure 2A shows the results for each emotion category, aggregated across the 60 individual participants, displayed as a color-coded face map. Warmer colors indicate higher numbers of participants, and cooler colors indicate lower numbers (color bar to right)—for example, in "happy," most participants perceived lip corner puller (AU12) to be associated with this emotion category (red regions around mouth corners; Figure S1A shows results as color-coded matrices). Figure 2B shows the results for the two-dimensional valence-arousal space (results normalized per face map for display purposes—see color bar to right; also, Figure S1B shows results as color-coded matrices).

Mapping facial expression signals of emotion categories and dimensions

Having modeled the facial expression signals that elicit the perception of emotion categories and of dimensions, we next examined whether they share certain facial movements by mapping the former onto the latter and examining their embedding. Specifically, for each emotion category, we computed the average similarity (i.e., correlation) between the facial expression signals (n = 60 models per category) and each facial expression signal across the valence-arousal dimensional space (n = 40 models per cell), thus producing a distribution of average correlation values across the valence-arousal space (Experiment 1,

Human facial expression signals are often multi-component, composed of different facial movements called action units (AUs)[14,15] that could each transmit information about (1) the specific emotion category (represented in red), (2) broad dimensions (represented in cyan), or (3) both categories and dimensions as a multiplexed signal (represented in magenta)—see Venn diagram. The face transmits the facial expression signal across the communication channel to the receiver, who then may perceive a message from the signal (here, successful decoding) based on their prior knowledge. We use this general framework of communication theory[28,32] to model the facial movements that are perceived to transmit emotion category and dimensional information.[19–27]

(B) Stimulus generation. On each experimental trial, a generative model of human facial movements[10] produced a random combination of facial movements by randomly selecting a subset of AUs—here, brow lowerer (AU4), nose wrinkler (AU9), and lip stretcher (AU20)—and assigning a random movement to each AU using six temporal parameters (see labels illustrating the solid black curve). The facial expression stimulus generated on this illustrative trial is shown at the bottom as four snapshots across time; the textured vector below shows the corresponding 3 (out of 42) randomly selected AUs. We displayed all facial expression stimuli on male and female face identities of the same ethnicity as participants (white).

(C) Perceptual task. Participants viewed the facial expression stimulus and interpreted it according to one of two pre-assigned perceptual tasks: (1) categorized according to one of the six classic emotion categories (red frame)—i.e., "happy," "surprise," "fear," "disgust," "anger," or "sad"—if and only if they perceived that the facial expression accurately represented one of the emotion categories or "don't know" if they did not or (2) the dimensions of valence (cyan frame)—"very negative" to "very positive"—and arousal—"low arousal" to "high arousal" on a 7-point scale in separate counterbalanced blocks. In this example trial, the participant perceived that the facial expression transmits the message "negative valence" (see black ellipse).

See also Video S1 for an illustration of the modeling procedure.

CellPress
OPEN ACCESS

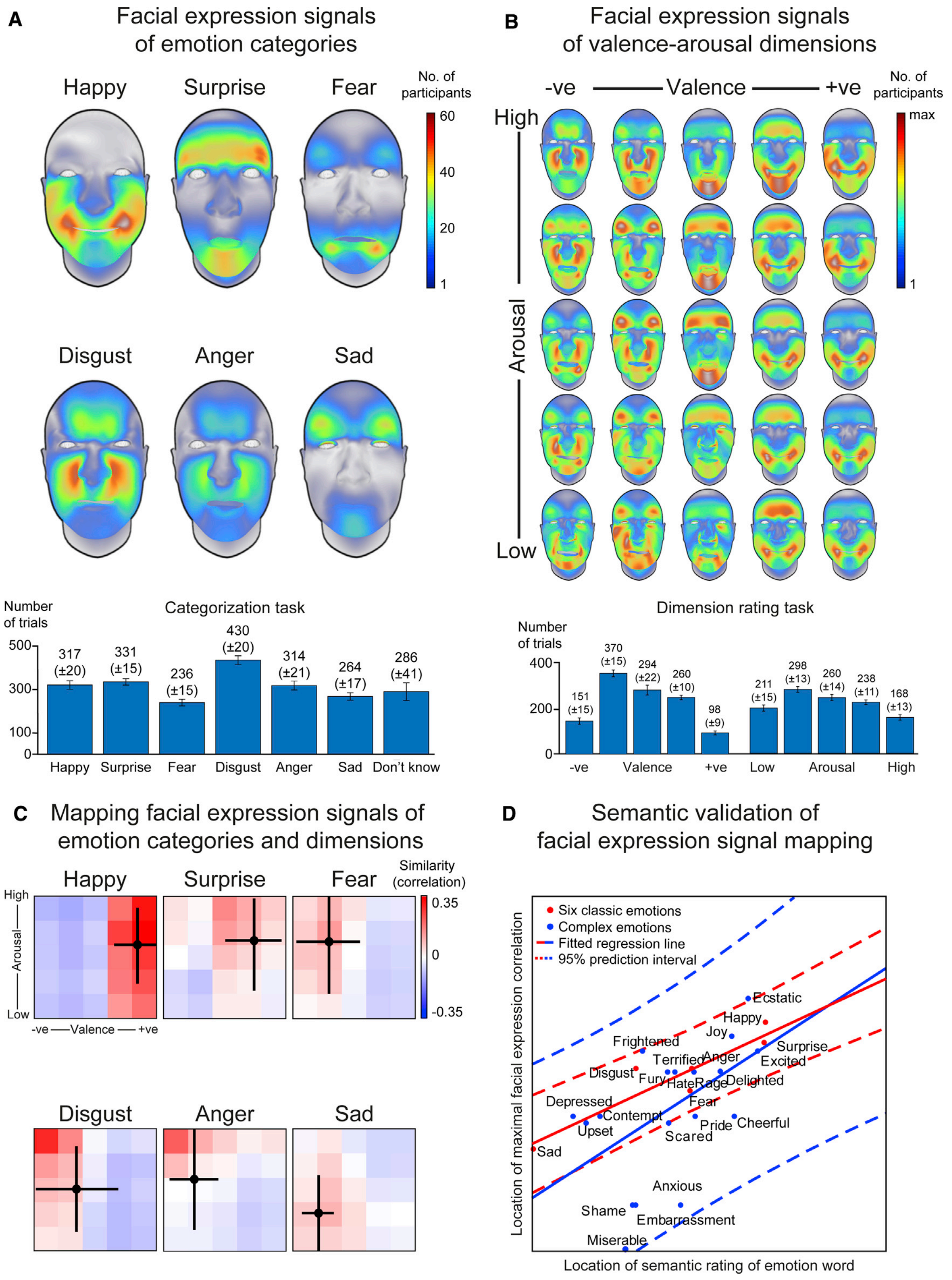

Figure 2. Mapping facial expression signals of emotion categories and dimensions

(A) Facial expression signals of emotion categories. Color-coded face maps show the facial expression signals (i.e., AU patterns) of the six classic emotions, summed across participants—see color bar to right (see also Figure S1A). Bar plots below show the average number of trials (±SEM) participants associated with each response option.

(B) Facial expression signals of valence and arousal dimensions. Color-coded face maps show the facial expression signals of the dimensions of valence and arousal, summed across participants and normalized per face map—see color bar to right (see also Figure S1B). Bar plots below show the average number of trials participants (±SEM) associated with each response option.

(legend continued on next page)

Facial expression mapping procedure—STAR Methods). Figure 2C shows the results for each emotion category as color-coded matrices. Red indicates higher similarity (i.e., positive correlations), and blue indicates lower similarity (negative correlations; see color bar to right). We then validated these mappings by comparing the location of the maximal correlation (saturated red squares, Figure 2C) with the semantic location of the corresponding emotion word[44] (black point with cross, Figure 2C) using linear regression. Results, shown in Figure 2D in red ($p = 0.0212$; two-tailed), confirmed that the mapping of the facial expressions of emotion categories onto the valence-arousal space corresponds with the semantic mapping of emotion category word (Experiment I, Validation of facial expression mappings—STAR Methods). Finally, we evaluated the generalizability of these results using a set of facial expression signals of 19 more complex emotions, including "delighted," "rage," and "terrified," derived using the same method to enable direct comparisons.[3] Results, shown in Figure 2D in blue ($p = 0.00418$, two-tailed; see also Figure S1C), further validate the facial expression mapping (Experiment I, Facial expression mapping procedure—STAR Methods). Together, these results show that facial expression signals that elicit the perception of emotion categories are embedded into those that elicit dimensional perceptions, suggesting that a latent set of shared AU jointly represent—i.e., multiplex—emotion category and dimensional information.

Experiment II: Measuring facial signal multiplexing of emotion categories and dimensions

To test this explicitly, we next disentangled the specific facial movements that serve this multiplexing role versus those that uniquely drive perceptions of emotion categories or dimensions (Figure 1A). We used an information-theoretic analysis called conditional mutual information (CMI),[11,45] which measures the relationship between two variables—here, an AU and the participants' emotion category responses—while accounting for the effects of a third variable—here, the participants' dimensional responses. For example, if the statistical relationship between lip corner puller (AU12) and the participants' emotion category responses is significantly high, this indicates that AU12 provides information about the participants' emotion category responses over and above that which it provides about their dimensional responses. Therefore, CMI enables precise characterization of the information that each AU provides about the receivers' responses and, thus, its capacity to jointly elicit—i.e., multiplex—emotion category and dimensional responses (Experiment II, Conditional Mutual Information analysis—STAR Methods). Using the same data-driven method (Figures 1B and 1C), a new set of participants (20 white Western, English-speaking, 10 females, 10 males; Participants, Experiment II—STAR Methods) each interpreted 1,200 newly randomly generated facial expressions (Experiment II, Stimuli—STAR Methods) according to the six classic emotion categories plus the dimensions of valence and arousal in three separate counterbalanced blocks in a within-subjects design (Experiment II, Perceptual task procedure—STAR Methods). We then used CMI to identify three types of facial signals: those perceived to (1) specifically transmit emotion category information; (2) specifically transmit dimensional information; and (3) jointly transmit—i.e., multiplex—categorical and dimensional information. Figure 3A, left panel, shows the results with each AU color coded accordingly (Venn diagram legend).

Results showed that most AUs involved in these perceptual tasks (i.e., 35/42; Figure S3) elicit the perception of emotion categories and dimensions (26/35 AUs; Figure 3A, magenta) with a subset specifically eliciting the perception of emotion categories (2/35 AUs; Figure 3A, red) or dimensions (7/35 AUs; Figure 3A, cyan). To characterize the specific emotion categories and dimensions that each facial movement is perceived to transmit, we used pointwise mutual information (PMI) (Experiment II, Conditional Mutual Information analysis—STAR Methods).[12] Figure 3A, center and right panels, show the results as color-coded face maps (see also Figure S3A). For example, chin raiser (AU17)—an emotion category signal (red)—elicits the perception of "disgust" and "sad." Unilateral lid tighteners (AU7L/R)—both dimensional signals (cyan)—elicit the perception of "negative valence" across high to low arousal. Nose wrinkler (AU9)—a multiplex signal (magenta)—elicits the perception of "disgust" and "anger" and "negative valence" across all high to low arousal. Figure 3B shows an example of the composition of such facial signals. Here, the facial expression, perceived as "disgust" and "negative valence, low arousal," comprises AUs that elicit the perception of "disgust"—chin raiser (AU17), upper lip raiser (AU10), lip corner depressor (AU15)—and of "negative valence" and "high arousal"—lid tightener (AU7), upper lip raiser (AU10), and lip corner depressor (AU15). Together, our results show that facial expressions comprise specific facial movement components that can jointly elicit the perception of—i.e., multiplex—emotion categories and dimensions.

Conclusions and future directions

Here, we have addressed the fundamental question of how facial expressions can achieve the complex signaling task of communicating broad-plus-specific emotion category and dimensional information (Figure 1A).[28,29] Across two main experiments, we used the classic data-driven method of reverse correlation to agnostically generate facial expressions—i.e., random combinations of AUs—and used the perceiver's perceptual responses plus information-theoretic analysis tools[11,12,45] to isolate the specific facial movements that elicit the perception of specific

(C) Mapping facial expression signals of emotion categories and dimensions. Each subplot shows, for each emotion category, the projection patterns of their facial expression signals onto those of valence and arousal dimensions, measured using correlation. Red represents high facial expression signal similarity (positive correlation); blue represents high dissimilarity (negative correlation—see color bar on right). Black crosses show the average valence and arousal rating of the emotion category word with standard deviation.[44]

(D) Semantic validation of facial expression signal mapping. A regression analysis comparing the location of the maximum facial expression signal correlation with the semantic location of the emotion word showed close correspondence (shown in red), thus validating the facial expression signal mappings. Analysis with a new set of facial expression signals of 19 of complex emotions[3] (shown in blue) provided further validation (Figure S1C; Facial expression mapping procedure—STAR Methods).

CellPress
OPEN ACCESS

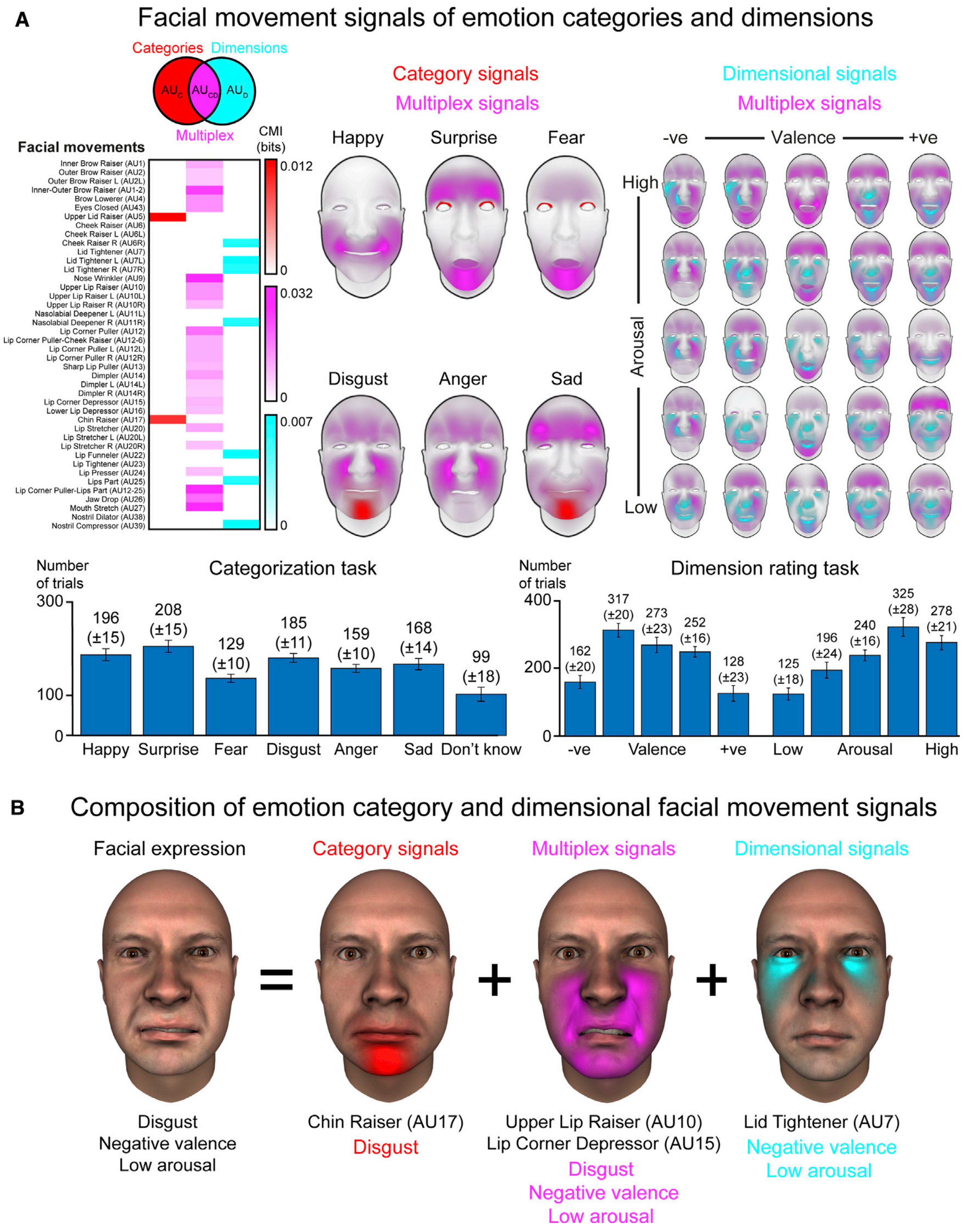

Figure 3. Facial movements perceived to transmit emotion category and dimensional information

(A) Facial movement signals of emotion categories and dimensions. The left panel shows each individual facial movement (i.e., AU; see labels on y axis) color coded according to whether it elicits the perception of emotion categories, dimensions, or both (see Venn diagram). Color saturation represents the CMI value, averaged across participants (see color bars to right). Most AUs comprise multiplex signals—i.e., perceived to transmit emotion category and dimensional information (represented by magenta)—with a subset perceived to transmit either emotion category (represented by red) or dimensional information (represented by cyan). Color-coded face maps in center and right show the specific emotion categories and dimensions each AU is perceived to transmit—for example, chin raiser (AU17), shown in red, elicits the perception of the emotion categories disgust and sad and nose wrinkler (AU9), shown in magenta, elicits the perception of disgust and anger, plus dimensional information (see also Figure S3A for a detailed breakdown). Bar charts below show the average number of trials (±SEM) participants associated with each response option.

(B) Composition of emotion category and dimensional facial movement signals. The illustrative example shows the composition of facial movements that are perceived to transmit emotion category and dimensional information in a facial expression.

emotion categories and/or broad dimensions (Figures 1B and 1C). We found that a latent set of shared facial movements can jointly elicit the perception of—i.e., multiplex—emotion categories and dimensions, with a subset eliciting perceptions of categories or dimensions (Figures 2 and 3). Our results suggest that facial expressions can drive perceptions of broad-plus-specific emotion messages via multiplexed facial signals. We now examine the implications of these findings.

We examined the critical stimulus-response relationship to identify the specific facial movements that drive emotion perception responses in receivers, thus providing a critical explanatory element that goes beyond accounts in which receiver responses are detached from the stimulus features that drive them.[6,7,46,47] Specifically, our results underline the close link between emotion category and dimensional perception by revealing their common signaling basis.[48] In contrast to some theoretical accounts,[49,50] our results suggest that facial expressions that drive emotion category perceptions are not distinct but instead structured by underlying signals of broad dimensions, mirroring existing findings.[51–53] This latent structure of multiplexed signaling could facilitate adaptive response—for example, broad dimensional signals could engage generalized approach versus avoid mechanisms[54] while category-specific signals could refine the message perceived and subsequent behavioral responses.[13]

These results thus raise the question of how such multiplexed signals are processed—for example, emotion category and dimensional information could be processed separately and asynchronously, with one informing the other (i.e., serving as priors). Our results suggest that broad-to-specific processing structure over time[7,13,55] because facial signals of dimensional information can predict specific emotion categories, but not vice versa (but see also Giordano et al.[56] for emotion vocalization processing). Broad dimensional information could also be more robust to degradation in the communication channel[57] and serve distal communication. Alternatively, such information could be processed synchronously, either separately in parallel or simultaneously, to produce a more refined percept. Task demands could also modulate the contribution of emotion category and dimension information by filtering out task-irrelevant information[24,55]—for example, where prioritizing categorical information over dimensional information improves performance.[7,58] Similarly, restricting access to language and/or conceptual knowledge could diminish one type of information while leaving the other intact.[59–61] Future work will examine these possibilities by tracing the dynamic processing of facial signals in the brain.[24,35,62]

Central to our finding of facial signal multiplexing is an information-theoretic analysis that goes beyond pairwise statistical methods to measure triplewise relationships—here, between AUs, emotion category responses, and dimensional responses.[35] We anticipate that such methods will become increasingly important in further understanding the complexities of human multimodal and multi-component signaling,[63–65] including their structural features.[9,66] Our results show that threat-related perceptions, particularly of anger, are elicited by a broader variety of facial signals than less threatening perceptions, which could reflect both higher signal degeneracy—different signals elicit similar perceptions—and redundancy—similar signals elicit similar perceptions (e.g., nose wrinkler [AU9] and upper lip raiser [AU10]—physically similar facial movements—each elicit anger and disgust perceptions). Such signal design features are particularly important for costly-to-miss threat messages within real-world noisy visual environments. Future work will address these questions using our methodological framework.

By using a paradigm that exploits the close symbiotic relationship between signal production and perception,[32,67–69] our results offer potential insights into facial expression production. As predicted by general accounts of communication,[19,28,29,66,70] human facial expression decoding, perceptual expertise, and conceptual knowledge,[71,72] the facial movements that elicit emotion perceptions likely comprise a subset of those that are produced. Future work will examine the precise relationship between facial movement production and perception to better understand the nature and function of human facial expressions.

Finally, facial expressions perceived in the real world are typically displayed alongside other sources of information—e.g., the expresser's identity, ethnicity, sex, gender, age, and culture; their voice and body movements; the nature of the interaction, social context, and scenery; and the communication channel across which signals are transmitted (e.g., proximal versus distal and clear versus occluded)—including dynamic changes that unfold over time. The perceiver's conceptual and cultural knowledge, expectations, and goals can also influence which information is attended to, extracted, and interpreted (e.g., see Archambault et al.,[73] Brooks and Freeman,[74] and Schyns and Rodet;[75] see Nisbett and Masuda[76] for a review). Understanding how each of these complex sources of information contribute to emotion perception (e.g., see Schyns et al.,[72] Hess et al.,[77] Gill et al.,[78] and Hehman et al.[79]) remains a centrally important empirical challenge. Using increasingly realistic generative models of faces,[43] scenes,[80] bodies,[81,82] and voices,[83] plus virtual reality technologies and new statistical tools,[11,35] our future work will address these major challenges. Relatedly, our results are based on white Western participants interpreting facial expressions displayed by same-ethnicity faces, using commonly used English-language emotion terms. Future work will prioritize examining whether these results generalize to or vary across other cultures, face identities, and languages (e.g., see Jack et al.,[41] Marsh et al.,[84] Elfenbein,[85] and Dailey et al.[86]).

In sum, our results provide new insights into the ontology and system of facial expression communication and present a methodological framework that can generate a richer account of human communication.

STAR★METHODS

Detailed methods are provided in the online version of this paper and include the following:

- KEY RESOURCES TABLE
- RESOURCE AVAILABILITY
  - Lead contact
  - Materials availability
  - Data and code availability
- EXPERIMENTAL MODEL AND SUBJECT DETAILS
  - Participants
  - Screening questionnaire
- METHOD DETAILS
  - Experiment I

CellPress
OPEN ACCESS

- Experiment II
- QUANTIFICATION AND STATISTICAL ANALYSIS
  - Experiment I
  - Experiment II

SUPPLEMENTAL INFORMATION

Supplemental information can be found online at https://doi.org/10.1016/j.cub.2021.10.035.

ACKNOWLEDGMENTS

This work was supported by the China Scholarship Council (CSC) (101706070134) awarded to M.L.; the Chinese Scholarship Council (201306270029), the Leverhulme Fellowship (ECF-2020-401), and the Lord Kelvin/Adam Smith Fellowship (201277) awarded to C.C.; the Wellcome Trust (214120/Z/18/Z) awarded to R.A.A.I.; the European Research Council (ERC) under the European Union's Horizon 2020 research and innovation programme (grant agreement FACESYNTAX no. 759796), British Academy (SG113332 and SG171783), and Economic and Social Research Council (ES/K001973/1 and ES/K00607X/1) awarded to R.E.J.; and the Wellcome Trust (Senior Investigator Award, UK; 107802) and the Multidisciplinary University Research Initiative/Engineering and Physical Sciences Research Council (USA, UK; 172046-01) awarded to P.G.S. The funders had no role in the study design, data collection or data analysis, decision to publish, or preparation of the manuscript.

AUTHOR CONTRIBUTIONS

Conceptualization, M.L., R.E.J., and P.G.S.; methodology, Y.D., R.A.A.I., P.G.S., O.G.B.G., R.E.J., and M.L.; software, P.G.S. and O.G.B.G.; formal analysis, Y.D., M.L., and R.A.A.I.; investigation, M.L. and C.C.; writing—original draft, M.L., R.E.J., and P.G.S.; writing—review & editing, M.L., R.E.J., R.A.A.I., C.C., and P.G.S.; visualization, M.L., R.E.J., and O.G.B.G.; supervision, R.E.J. and P.G.S.; funding acquisition, R.E.J. and P.G.S.

DECLARATION OF INTERESTS

The authors declare no competing interests.

Received: June 8, 2021
Revised: September 7, 2021
Accepted: October 14, 2021
Published: November 11, 2021

REFERENCES

1. Darwin, C. (2015). The Expression of the Emotions in Man and Animals (University of Chicago).
2. Du, S., Tao, Y., and Martinez, A.M. (2014). Compound facial expressions of emotion. Proc. Natl. Acad. Sci. USA *111*, E1454–E1462.
3. Jack, R.E., Sun, W., Delis, I., Garrod, O.G.B., and Schyns, P.G. (2016). Four not six: revealing culturally common facial expressions of emotion. J. Exp. Psychol. Gen. *145*, 708–730.
4. Mortillaro, M., Mehu, M., and Scherer, K.R. (2011). Subtly different positive emotions can be distinguished by their facial expressions. Soc. Psychol. Personal. Sci. *2*, 262–271.
5. Keltner, D. (1996). Evidence for the distinctness of embarrassment, shame, and guilt: a study of recalled antecedents and facial expressions of emotion. Cogn. Emotion *10*, 155–172.
6. Cowen, A.S., and Keltner, D. (2020). What the face displays: mapping 28 emotions conveyed by naturalistic expression. Am. Psychol. *75*, 349–364.
7. Mendolia, M. (2007). Explicit use of categorical and dimensional strategies to decode facial expressions of emotion. J. Nonverbal Behav. *31*, 57–75.
8. Mehu, M., and Scherer, K.R. (2015). Emotion categories and dimensions in the facial communication of affect: an integrated approach. Emotion *15*, 798–811.
9. Hebets, E.A., Barron, A.B., Balakrishnan, C.N., Hauber, M.E., Mason, P.H., and Hoke, K.L. (2016). A systems approach to animal communication. Proc. Biol. Sci. *283*, 20152889.
10. Yu, H., Garrod, O.G.B., and Schyns, P.G. (2012). Perception-driven facial expression synthesis. Comput. Graph. *36*, 152–162.
11. Ince, R.A.A., Giordano, B.L., Kayser, C., Rousselet, G.A., Gross, J., and Schyns, P.G. (2017). A statistical framework for neuroimaging data analysis based on mutual information estimated via a gaussian copula. Hum. Brain Mapp. *38*, 1541–1573.
12. Bouma, G. (2009). Normalized (pointwise) mutual information in collocation extraction. Proc. GSCL, pp. 31–40.
13. Jack, R.E., Garrod, O.G.B., and Schyns, P.G. (2014). Dynamic facial expressions of emotion transmit an evolving hierarchy of signals over time. Curr. Biol. *24*, 187–192.
14. Ekman, P., Friesen, W., and Hagar, J.C. (1978). Facial Action Coding System: Investigator's Guide (Research Nexus). Palo Alto *3*, 5.
15. Hjortsjö, C.-H. (1969). Man's Face and Mimic Language (Studentlitteratur).
16. Ekman, P., Friesen, W.V., and Hager, J.C. (2002). Facial Action Coding System: The Manual on CD ROM (A Human Face), pp. 77–254.
17. Crivelli, C., and Fridlund, A.J. (2018). Facial displays are tools for social influence. Trends Cogn. Sci. *22*, 388–399.
18. Frith, C. (2009). Role of facial expressions in social interactions. Philos. Trans. R. Soc. Lond. B Biol. Sci. *364*, 3453–3458.
19. Tinbergen, N. (1948). Social releasers and the experimental method required for their study. Wilson Bull. *60*, 6–51.
20. Mangini, M.C., and Biederman, I. (2004). Making the ineffable explicit: estimating the information employed for face classifications. Cogn. Sci. *28*, 209–226.
21. Murray, R.F. (2011). Classification images: a review. J. Vis. *11*, 2.
22. Hubel, D.H., and Wiesel, T.N. (1959). Receptive fields of single neurones in the cat's striate cortex. J. Physiol. *148*, 574–591.
23. Nishimoto, S., Ishida, T., and Ohzawa, I. (2006). Receptive field properties of neurons in the early visual cortex revealed by local spectral reverse correlation. J. Neurosci. *26*, 3269–3280.
24. Zhan, J., Ince, R.A.A., van Rijsbergen, N., and Schyns, P.G. (2019). Dynamic construction of reduced representations in the brain for perceptual decision behavior. Curr. Biol. *29*, 319–326.e4.
25. Volterra, V., and Whittaker, E.T. (1959). Theory of Functionals and of Integral and Integro-differential Equations (Dover).
26. Wiener, N. (1958). Nonlinear Problems in Random Theory (Technology Press of Massachusetts Institute of Technology).
27. Thompson, W.B., Owen, J.C., de St. Germain, H.J., Stark, S.R., and Henderson, T.C. (1999). Feature-based reverse engineering of mechanical parts. IEEE Trans. Robot. Autom. *15*, 57–66.
28. Shannon, C.E. (1948). A mathematical theory of communication. Bell Syst. Tech. J. *27*, 379–423.
29. Bradbury, J.W., and Vehrencamp, S.L. (1998). Principles of Animal Communication (Sinauer Associates).
30. Dukas, R. (1998). Cognitive Ecology: The Evolutionary Ecology of Information Processing and Decision Making (University of Chicago).
31. Slater, P.J., Halliday, T.R., and Barrett, P. (1994). Behaviour and Evolution (Cambridge University).
32. Scott-Phillips, T.C. (2008). Defining biological communication. J. Evol. Biol. *21*, 387–395.
33. Barrett, L.F., Adolphs, R., Marsella, S., Martinez, A.M., and Pollak, S.D. (2019). Emotional expressions reconsidered: challenges to inferring emotion from human facial movements. Psychol. Sci. Public Interest *20*, 1–68.

34. Jack, R.E., and Schyns, P.G. (2017). Toward a social psychophysics of face communication. Annu. Rev. Psychol. *68*, 269–297.
35. Schyns, P.G., Zhan, J., Jack, R.E., and Ince, R.A.A. (2020). Revealing the information contents of memory within the stimulus information representation framework. Philos. Trans. R. Soc. Lond. B Biol. Sci. *375*, 20190705.
36. Krakauer, J.W., Ghazanfar, A.A., Gomez-Marin, A., MacIver, M.A., and Poeppel, D. (2017). Neuroscience needs behavior: correcting a reductionist bias. Neuron *93*, 480–490.
37. Wu, M.C.-K., David, S.V., and Gallant, J.L. (2006). Complete functional characterization of sensory neurons by system identification. Annu. Rev. Neurosci. *29*, 477–505.
38. Naselaris, T., Kay, K.N., Nishimoto, S., and Gallant, J.L. (2011). Encoding and decoding in fMRI. Neuroimage *56*, 400–410.
39. Kriegeskorte, N., and Douglas, P.K. (2018). Cognitive computational neuroscience. Nat. Neurosci. *21*, 1148–1160.
40. Rychlowska, M., Jack, R.E., Garrod, O.G.B., Schyns, P.G., Martin, J.D., and Niedenthal, P.M. (2017). Functional smiles: tools for love, sympathy, and war. Psychol. Sci. *28*, 1259–1270.
41. Jack, R.E., Garrod, O.G.B., Yu, H., Caldara, R., and Schyns, P.G. (2012). Facial expressions of emotion are not culturally universal. Proc. Natl. Acad. Sci. USA *109*, 7241–7244.
42. Gosselin, F., and Schyns, P.G. (2003). Superstitious perceptions reveal properties of internal representations. Psychol. Sci. *14*, 505–509.
43. Zhan, J., Garrod, O.G.B., van Rijsbergen, N., and Schyns, P.G. (2019). Modelling face memory reveals task-generalizable representations. Nat. Hum. Behav. *3*, 817–826.
44. Warriner, A.B., Kuperman, V., and Brysbaert, M. (2013). Norms of valence, arousal, and dominance for 13,915 English lemmas. Behav. Res. Methods *45*, 1191–1207.
45. Cover, T.M. (1999). Elements of Information Theory (John Wiley & Sons).
46. Bimler, D., and Kirkland, J. (2001). Categorical perception of facial expressions of emotion: evidence from multidimensional scaling. Cogn. Emotion *15*, 633–658.
47. Fujimura, T., Matsuda, Y.-T., Katahira, K., Okada, M., and Okanoya, K. (2012). Categorical and dimensional perceptions in decoding emotional facial expressions. Cogn. Emotion *26*, 587–601.
48. Medin, D.L., Goldstone, R.L., and Gentner, D. (1993). Respects for similarity. Psychol. Rev. *100*, 254–278.
49. Ekman, P. (1992). An argument for basic emotions. Cogn. Emotion *6*, 169–200.
50. Tracy, J.L., and Randles, D. (2011). Four models of basic emotions: a review of Ekman and Cordaro, Izard, Levenson, and Panksepp and Watt. Emot. Rev. *3*, 397–405.
51. Cowen, A.S., and Keltner, D. (2017). Self-report captures 27 distinct categories of emotion bridged by continuous gradients. Proc. Natl. Acad. Sci. USA *114*, E7900–E7909.
52. Fontaine, J.R.J., Scherer, K.R., Roesch, E.B., and Ellsworth, P.C. (2007). The world of emotions is not two-dimensional. Psychol. Sci. *18*, 1050–1057.
53. Barrett, L.F. (1998). Discrete emotions or dimensions? The role of valence focus and arousal focus. Cogn. Emotion *12*, 579–599.
54. Krieglmeyer, R., Deutsch, R., De Houwer, J., and De Raedt, R. (2010). Being moved: valence activates approach-avoidance behavior independently of evaluation and approach-avoidance intentions. Psychol. Sci. *21*, 607–613.
55. van Rijsbergen, N.J., and Schyns, P.G. (2009). Dynamics of trimming the content of face representations for categorization in the brain. PLoS Comput. Biol. *5*, e1000561.
56. Giordano, B.L., Whiting, C., Kriegeskorte, N., Kotz, S.A., Gross, J., and Belin, P. (2021). The representational dynamics of perceived voice emotions evolve from categories to dimensions. Nat. Hum. Behav. *5*, 1203–1213.
57. Smith, F.W., and Schyns, P.G. (2009). Smile through your fear and sadness: transmitting and identifying facial expression signals over a range of viewing distances. Psychol. Sci. *20*, 1202–1208.
58. Miellet, S., Caldara, R., and Schyns, P.G (2011). Local Jekyll and global Hyde: the dual identity of face identification. Psychol. Sci. *22*, 1518–1526.
59. Roberson, D., and Davidoff, J. (2000). The categorical perception of colors and facial expressions: the effect of verbal interference. Mem. Cognit. *28*, 977–986.
60. Shablack, H., and Lindquist, K.A. (2019). The role of language in emotional development. In Handbook of Emotional Development, V. LoBue, K. Pérez-Edgar, and K.A. Buss, eds. (Springer International), pp. 451–478.
61. Widen, S.C. (2013). Children's interpretation of facial expressions: the long path from valence-based to specific discrete categories. Emot. Rev. *5*, 72–77.
62. Schyns, P.G., Petro, L.S., and Smith, M.L. (2007). Dynamics of visual information integration in the brain for categorizing facial expressions. Curr. Biol. *17*, 1580–1585.
63. Holler, J., and Levinson, S.C. (2019). Multimodal language processing in human communication. Trends Cogn. Sci. *23*, 639–652.
64. Keltner, D., and Cordaro, D.T. (2017). Understanding multimodal emotional expressions: Recent advances in basic emotion theory. In The Science of Facial Expression, J.-M. Fernández-Dols, and J.A. Russell, eds. (Oxford University), pp. 57–75.
65. Higham, J.P., and Hebets, E.A. (2013). An introduction to multimodal communication. Behav. Ecol. Sociobiol. *67*, 1381–1388.
66. Edelman, G.M., and Gally, J.A. (2001). Degeneracy and complexity in biological systems. Proc. Natl. Acad. Sci. USA *98*, 13763–13768.
67. Guilford, T., and Dawkins, M.S. (1991). Receiver psychology and the evolution of animal signals. Anim. Behav. *42*, 1–14.
68. Darwin, C. (2009). In The Expression of the Emotions in Man and Animals, F. Darwin, ed. (Cambridge University).
69. Arak, A., and Enquist, M. (1993). Hidden preferences and the evolution of signals. Philos. Trans. R. Soc. B: Biol. Sci. *340*, 207–213.
70. McGregor, P.K. (2005). Animal Communication Networks (Cambridge University).
71. Tanaka, J.W., and Taylor, M. (1991). Object categories and expertise: is the basic level in the eye of the beholder? Cognit. Psychol. *23*, 457–482.
72. Schyns, P.G., Goldstone, R.L., and Thibaut, J.-P. (1998). The development of features in object concepts. Behav. Brain Sci. *21*, 1–17, discussion 17–54.
73. Archambault, A., O'Donnell, C., and Schyns, P.G. (1999). Blind to object changes: when learning the same object at different levels of categorization modifies its perception. Psychol. Sci. *10*, 249–255.
74. Brooks, J.A., and Freeman, J.B. (2018). Conceptual knowledge predicts the representational structure of facial emotion perception. Nat. Hum. Behav. *2*, 581–591.
75. Schyns, P.G., and Rodet, L. (1997). Categorization creates functional features. J. Exp. Psychol. Learn. Mem. Cogn. *23*, 681–696.
76. Nisbett, R.E., and Masuda, T. (2003). Culture and point of view. Proc. Natl. Acad. Sci. USA *100*, 11163–11170.
77. Hess, U., Adams, R.B., Jr., and Kleck, R.E. (2009). The face is not an empty canvas: how facial expressions interact with facial appearance. Philos. Trans. R. Soc. Lond. B Biol. Sci. *364*, 3497–3504.
78. Gill, D., Garrod, O.G., Jack, R.E., and Schyns, P.G. (2014). Facial movements strategically camouflage involuntary social signals of face morphology. Psychol. Sci. *25*, 1079–1086.
79. Hehman, E., Stolier, R.M., Freeman, J.B., Flake, J.K., and Xie, S.Y. (2019). Toward a comprehensive model of face impressions: what we know, what we do not, and paths forward. Soc. Personal. Psychol. Compass *13*, e12431.
80. Zhu, S.-C., and Mumford, D. (2007). A Stochastic Grammar of Images (Now Publishers).

CellPress
OPEN ACCESS

81. Ishii, R., Katayama, T., Higashinaka, R., and Tomita, J. (2018). Generating body motions using spoken language in dialogue. In Proceedings of the 18th International Conference on Intelligent Virtual Agents IVA '18 (Association for Computing Machinery), pp. 87–92.
82. Lee, J., and Marsella, S. (2006). Nonverbal behavior generator for embodied conversational agents. In Intelligent Virtual Agents Lecture Notes in Computer Science, J. Gratch, M. Young, R. Aylett, D. Ballin, and P. Olivier, eds. (Springer), pp. 243–255.
83. Arias, P., Rachman, L., Liuni, M., and Aucouturier, J.-J. (2021). Beyond correlation: acoustic transformation methods for the experimental study of emotional voice and speech. Emot. Rev. *13*, 12–24.
84. Marsh, A.A., Elfenbein, H.A., and Ambady, N. (2003). Nonverbal "accents": cultural differences in facial expressions of emotion. Psychol. Sci. *14*, 373–376.
85. Elfenbein, H.A. (2013). Nonverbal dialects and accents in facial expressions of emotion. Emot. Rev. *5*, 90–96.
86. Dailey, M.N., Joyce, C., Lyons, M.J., Kamachi, M., Ishi, H., Gyoba, J., and Cottrell, G.W. (2010). Evidence and a computational explanation of cultural differences in facial expression recognition. Emotion *10*, 874–893.
87. De Leersnyder, J., Mesquita, B., and Kim, H.S. (2011). Where do my emotions belong? A study of immigrants' emotional acculturation. Pers. Soc. Psychol. Bull. *37*, 451–463.
88. Roberson, D., Damjanovic, L., and Kikutani, M. (2010). Show and tell: the role of language in categorizing facial expression of emotion. Emot. Rev. *2*, 255–260.
89. Chua, H.F., Boland, J.E., and Nisbett, R.E. (2005). Cultural variation in eye movements during scene perception. Proc. Natl. Acad. Sci. USA *102*, 12629–12633.
90. Jack, R.E. (2013). Culture and facial expressions of emotion. Vis. Cogn. *21*, 1248–1286.
91. McKone, E., Dawel, A., Robbins, R.A., Shou, Y., Chen, N., and Crookes, K. (2021). Why the other-race effect matters: Poor recognition of other-race faces impacts everyday social interactions. Br. J. Psychol. Published online May 19, 2021. https://doi.org/10.1111/bjop.12508.
92. Russell, J.A. (1980). A circumplex model of affect. J. Pers. Soc. Psychol. *39*, 1161–1178.
93. Schubert, E. (1999). Measuring emotion continuously: validity and reliability of the two-dimensional emotion-space. Aust. J. Psychol. *51*, 154–165.
94. Bradley, M.M., and Lang, P.J. (1999). Affective norms for English words (ANEW): instruction manual and affective ratings. Technical report C-1 (The Center for Research in Psychophysiology, University of Florida).
95. Hall, E.T. (1966). The Hidden Dimension (Doubleday).
96. Russell, J.A. (1994). Is there universal recognition of emotion from facial expression? A review of the cross-cultural studies. Psychol. Bull. *115*, 102–141.
97. Chen, C., Crivelli, C., Garrod, O.G.B., Schyns, P.G., Fernández-Dols, J.-M., and Jack, R.E. (2018). Distinct facial expressions represent pain and pleasure across cultures. Proc. Natl. Acad. Sci. USA *115*, E10013–E10021.
98. Chen, C., Hensel, L.B., Duan, Y., Ince, R.A.A., Garrod, O.G.B., Beskow, J., Jack, R.E., and Schyns, P.G. (2019). Equipping social robots with culturally-sensitive facial expressions of emotion using data-driven methods. In 2019 14th IEEE International Conference on Automatic Face Gesture Recognition (FG 2019) (IEEE), pp. 1–8.
99. Ekman, P., Sorenson, E.R., and Friesen, W.V. (1969). Pan-cultural elements in facial displays of emotion. Science *164*, 86–88.
100. Martin, J., Rychlowska, M., Wood, A., and Niedenthal, P. (2017). Smiles as multipurpose social signals. Trends Cogn. Sci. *21*, 864–877.
101. Parkinson, B. (2005). Do facial movements express emotions or communicate motives? Pers. Soc. Psychol. Rev. *9*, 278–311.
102. Susskind, J.M., Lee, D.H., Cusi, A., Feiman, R., Grabski, W., and Anderson, A.K. (2008). Expressing fear enhances sensory acquisition. Nat. Neurosci. *11*, 843–850.
103. Bavelas, J.B., Coates, L., and Johnson, T. (2000). Listeners as co-narrators. J. Pers. Soc. Psychol. *79*, 941–952.
104. Ekman, P., and Oster, H. (1979). Facial expressions of emotion. Annu. Rev. Psychol. *30*, 527–554.
105. Oosterhof, N.N., and Todorov, A. (2008). The functional basis of face evaluation. Proc. Natl. Acad. Sci. USA *105*, 11087–11092.
106. Hess, U., Philippot, P., and Blairy, S. (1998). Facial reactions to emotional facial expressions: affect or cognition? Cogn. Emotion *12*, 509–531.
107. Nichols, T.E., and Holmes, A.P. (2002). Nonparametric permutation tests for functional neuroimaging: a primer with examples. Hum. Brain Mapp. *15*, 1–25.
108. Aviezer, H., Hassin, R.R., Ryan, J., Grady, C., Susskind, J., Anderson, A., Moscovitch, M., and Bentin, S. (2008). Angry, disgusted, or afraid? Studies on the malleability of emotion perception. Psychol. Sci. *19*, 724–732.
109. Widen, S.C., Russell, J.A., and Brooks, A. (2004). Anger and disgust: discrete or overlapping categories. In 2004 APS Annual Convention (Boston College).
110. Jack, R.E., Blais, C., Scheepers, C., Schyns, P.G., and Caldara, R. (2009). Cultural confusions show that facial expressions are not universal. Curr. Biol. *19*, 1543–1548.

STAR★METHODS

KEY RESOURCES TABLE

REAGENT or RESOURCE	SOURCE	IDENTIFIER
Deposited data		
Raw and analyzed data	This paper	https://doi.org/10.17632/dr853shk56.3
Software and algorithms		
MATLAB R2016b & R2020b	MathWorks	RRID: SCR_001622
Psychtoolbox-3	http://psychtoolbox.org/	RRID: SCR_002881
Custom Code for analyses	This paper	https://doi.org/10.17632/dr853shk56.3

RESOURCE AVAILABILITY

Lead contact

Further information and requests for resources should be directed to and will be fulfilled by the Lead Contact, Rachael E. Jack (rachael.jack@glasgow.ac.uk)

Materials availability

This study did not generate new unique reagents.

Data and code availability

Raw and analyzed data reported in this study are deposited in Mendeley Data, https://doi.org/10.17632/dr853shk56.2. Custom code for analyses is deposited in Mendeley Data, https://doi.org/10.17632/dr853shk56.3. Custom code for modeling, experiment, and visualization are available by request to the Lead Contact.

EXPERIMENTAL MODEL AND SUBJECT DETAILS

Participants

Experiment I

For the emotion categorization task, we used an existing dataset comprising 60 participants (white, Western, English-speaking, 31 females, 29 males, mean age = 22 years, SD = 1.71 years; see Jack et al.[13]). For the valence and arousal dimensional rating tasks, we recruited a separate set of 40 same-culture, ethnicity, age, and sex-balanced participants (white, Western, English-speaking, 20 females, 20 males, mean age = 21 years, SD = 2.52 years). All 100 participants had minimal experience of/exposure to non-Western cultures (as assessed by questionnaire, see Screening Questionnaire—STAR Methods, e.g., see De Leersnyder et al.[87]), normal or corrected-to-normal vision, and without any emotion-related atypicalities (Autism Spectrum Disorder, depression, anxiety), learning difficulties (e.g., dyslexia), synesthesia, or disorders of face perception (e.g., prosopagnosia) as per self-report. All participants gave written informed consent prior to testing and received a standard rate of £6/h for their participation. The University of Glasgow College of Science and Engineering Ethics Committee provided ethical approval (Ref: 300180112). All experiments conform to the British Psychological Society's Code of Human Research Ethics.

Experiment II

We recruited a further set of 20 new participants (white, Western, English-speaking, 10 females, 10 males, mean age = 20.3 years, SD = 2.23 years) using the same criteria as described above. All participants gave written informed consent prior to testing and received a standard rate of £6/h for their participation. The University of Glasgow College of Science and Engineering Ethics Committee provided ethical approval (Ref: 300180277). All experiments conform to the British Psychological Society's Code of Human Research Ethics.

Screening questionnaire

Given that culture and linguistic background is a known source of variance in perception (e.g., see Shablack and Lindquist,[60] Nisbett and Masuda,[76] Roberson et al.,[88] Chua et al.,[89] and Jack[90] for reviews), and that differences between the ethnicity of face stimuli and participants can modulate social face perception (e.g., see McKone et al.[91] for a review), we controlled these factors by including a sample of same-culture (Western) and same-ethnicity (white) English-speaking participants—a population in which the six classic emotion categories and the dimensions of valence and arousal are well-established constructs.[90,92–94] Future work will further examine whether and how such factors influence the perception of facial expressions as categorical and/or dimensional signals.

To control for the effects of exposure to/experience of other cultures,[87] each potential participant completed the following questionnaire. We only selected individuals who answered ‘no’ to all questions for participation in the experiments:

1. Have you ever lived in non-Western* country before (e.g., on a gap year, summer work, move due parental employment)?
2. How many weeks have you spent in a non-Western country (e.g., on vacation)?
3. Have you ever dated or had a very close friendship with a non-Western person?
4. Have you ever been involved with any non-Western culture societies/groups?

*By Western groups/countries, we are referring to Europe (East and West), USA, Canada, United Kingdom, Australia, and New Zealand.

METHOD DETAILS

Experiment I

Stimuli

For both the emotion categorization task and the dimensional rating tasks, we generated facial expression stimuli using the same procedure as follows. We used a generative model of human facial movements,[10] which is comprised of a library of individual 3D facial action units (AUs)—i.e., the basic elements of human facial movements as detailed by the taxonomic Facial Action Coding System (FACS,[14] see also Hjortsjö[15]). Each AU in the generative model is derived from real humans, who are trained to accurately produce each individual AU on their face, captured using a stereoscopic system and their rendering verified by the trained AU producers (see Yu et al.[10]). Therefore, the generative model produces valid representations of real human facial movements and comprises no physiologically impossible facial movements (e.g., rotating the nose 90 degrees, sinking the eyeballs deep into the head, lowering the eyebrows below the eyes; see Yu et al.[10] for further details). To generate facial expression stimuli on each experimental trial, the generative model of facial movements pseudo-randomly selected a combination of AUs from a set of 42 Action Units (minimum = 1 AU, maximum = 4 AUs, median = 3 AUs selected across trials) and assigned a random movement to each AU using six temporal parameters—onset latency, acceleration, peak amplitude, peak latency, deceleration, offset latency (in Figure 1B, see labels describing the black temporal curve). These six temporal parameters enabled each AU to peak once during the stimulus time course while other parameters such as acceleration and amplitude could vary across the experiment, thus enabling exploration of dynamic properties while retaining experimental feasibility (see Yu et al.[10]). Future work will examine the relevance of multiple AU peaks—e.g., lip quivering, repeated eyebrow raising—in driving social and emotion perception.

For the emotion categorization task we generated 2400 random facial expressions—i.e., random combinations of dynamic AUs—and displayed each on one of 8 photorealistic face identities of real people of the same ethnicity as participants (white, 4 females, 4 males, mean age = 28 years, SD = 3.85 years), captured using a high-resolution 3D face capture system (see Yu et al.[10]), to control for the potential effects of other-ethnicity perception.[91] For the valence and arousal dimensional rating task, we generated a further 2400 random facial expressions and displayed each on the same face identities used in the emotion categorization task. For each participant, we randomly split the stimuli into two sets of 1200 facial expressions and assigned each stimulus set to one of the two rating tasks of valence and arousal. In both rating tasks, we included face identities of the same ethnicity as participants (white) to control for the potential effects of other-ethnicity perception.[91] Future work will examine whether and how the perceiver’s culture and the ethnicity of the face stimuli each influence the perception of facial expressions as categorical and/or dimensional signals. In experiments and tasks, we displayed all facial expression stimuli in the center of the participant’s visual field, on a black background, and on a 19-inch flat panel Dell monitor (Round Rock, Texas 78682, refresh rate of 60 Hz and resolution of 1024 × 1280). Participants used a chin rest to maintain a constant viewing distance, with stimuli subtending 14.25° (vertical) × 10.08°(horizontal) of visual angle in the emotion categorization task (average stimulus size 17.00 cm × 11.99 cm) and 13.74° × 8.65° of visual angle in the dimensional rating task (average stimulus size 19.54 cm × 12.28 cm), each reflecting the average size of a human face during typical social interaction in Western culture.[95]

Perceptual task procedure

Participants viewed a randomly generated facial expression on each trial and interpreted it according to one of two pre-assigned tasks in a between-subjects design: (1) categorize according to one of the six classic emotions—i.e., “happy,” “surprise,” “fear,” “disgust,” “anger” or “sad”—each well-established emotion concepts within Western culture, English-speaking populations (see Jack[90] for a review)—or (2) rate according to the dimensions of valence or arousal—each well-established dimensional concepts within Western culture, English-speaking populations[92–94]—in separate counterbalanced blocks. In the dimensional rating task, participants rated each facial expression stimulus according to (1) valence on a 7-point scale from “very negative” to “very positive,” and (2) arousal on a 7-point scale from “low arousal” to “high arousal.” In the emotion categorization task, participants categorized each facial expression stimulus according to one of the six classic emotions if, and only if, they perceived that the facial movement accurately represented that emotion message—i.e., corresponded with their prior knowledge of facial expressions of the emotion message. If the participant selected an emotion label, they also rated the intensity on a 5-point scale from “very weak” to “very strong.” If the participant perceived that the facial movement did not accurately represent any of the emotion messages, including if it represented a compound/blended emotion message such as “happily disgusted,”[2] they selected “don’t know.” Therefore, we explicitly used a behavioral task that does not force participants to select unrepresentative facial

expressions as representing emotion messages—i.e., building artificial relationships between stimuli and perceptions (e.g., see Russell[96] for discussion)—and thus enables participants to separate the facial expressions that are representative of these emotion messages from those that are not, based on their prior knowledge of the external world (see Jack et al.[3,41] and Chen et al.[97,98] for validation examples; see also Darwin[68] and Ekman et al.[99] for similar applications, but see also Russell[96] for discussion on task demands). Note that facial expressions observed in the real world do not necessarily reflect the internal states of expressers.[17,33,100,101] Thus, in modeling facial expressions of emotion messages—e.g., "disgust," or "negative valence, high arousal"—we do not assume that such messages necessarily reflect the internal emotional states of expressers. Similarly, although human facial movements can serve multiple functions—including displaying internal emotional states that can benefit both producers[102] and receivers,[1,32] communicating social messages to others[17,103,104] (e.g., back channeling), or serving physiological needs (e.g., sneezing, chewing, squinting)—here, we specifically examine the relationship between facial movement stimuli and receiver perceptual responses, not the relationship between internal emotion states and external facial displays.

In each experiment and all perceptual tasks, each facial expression played once for a duration of 1.25 s followed by a black screen. We instructed participants to respond quicky based on their first impressions and to use a mouse-operated Graphic User Interface (GUI) to register their responses. Participants could respond only after the facial expression stimulus had finished playing and had unlimited time to respond. After response, the next trial started. In the emotion categorization task, we randomized the order of the trials across the experiment for each participant. In the dimensional rating task, we blocked the two tasks of rating valence and arousal, counterbalanced the order of the blocks across participants, and randomized the order of the trials within each block for each participant. In both the emotion categorization task and the dimensional rating task, we divided the trials into separate sessions of 200 trials, with each session split into 4 sets of 50 trials and each set separated by a short break. After three consecutive sessions of 200 trials, participants took a required break of at least 1 hour. Note that the task included specific response options—i.e., the six classic emotion categories and dimensions of valence and arousal—rather than free response options or an extensive list of emotion categories and dimensions to avoid combinatorial explosions and the curse of dimensionality, thus enabling application of this data-driven method (e.g., see Jack and Schyns[34] for discussion). Future work will examine whether and how other socially relevant dimensions are transmitted by facial movements, their relative contributions to perceptual response outcomes, and their potential multiplexing with categorical information (e.g., see Fontaine et al.,[52] Oosterhof and Todorov,[105] and Hess et al.[106]).

Experiment II

Stimuli

In a separate within-subjects designed experiment, we generated a further 1200 random facial expressions using the same stimulus generation procedure described above (see Figures 1B and 1C, and Modeling facial signals of emotion information—STAR Methods). We displayed each facial expression on a randomly generated face identity of the same ethnicity as participants (white, 600 females, 600 males aged 20–40 years) using a face identity generator that is based on high resolution 3D captures of real people and has a high fidelity generative capacity (see Zhan et al.[43]). As with the other experiments, we included face identities of the same ethnicity as participants (white) to control for the potential effects of other-ethnicity perception. Future work will examine whether and how the perceiver's culture and the ethnicity of face stimuli each influence the perception of facial expressions as categorical and/or dimensional signals.

Perceptual task procedure

We used the same perceptual task procedure as in the two other experiments. Each participant viewed a randomly generated facial expression and interpreted it according to one of three pre-assigned tasks: (1) categorize according to one of the six classic emotions—i.e., "happy," "surprise," "fear," "disgust," "anger" or "sad"—if, and only if, the participant perceived that the facial expression accurately represented the emotion message, or "other" if they perceived that it did not, including blended/compound emotions;[2] (2) rate by valence on a 7-point scale from "very negative" to "very positive;" and (3) rate by arousal on a 7-point scale from "low arousal" to "high arousal." We used the same stimulus display and response conditions and as described above. Participants used a chin rest to maintain a constant viewing distance of 47cm, with stimuli subtending 14.42° (vertical) × 8.80° (horizontal) of visual angle (average stimulus size 11.89 cm × 7.23 cm), reflecting the average size of a human face during typical social interaction.[95] Each participant viewed the same 1200 facial expression in each of the three tasks, presented in random order within each task. We blocked the three tasks into two main blocks—emotion categorization and dimensional ratings—and counterbalanced the order of these two blocks across participants. Within the dimensional rating block, we further blocked and randomized the order of the valence and arousal tasks across participants. In each of the three tasks, we divided the trials and structured breaks in the same way as described above.

QUANTIFICATION AND STATISTICAL ANALYSIS

Experiment I

Facial expression modeling procedure

To model the facial expression signals that elicit the perception of emotion categories and, separately, dimensions, we used the non-parametric statistical method of mutual information (MI[11]), which measures the statistical dependence between two variables—here, an AU and the participant's emotion category perceptual response—without assumptions about the linear or non-linear nature of the relationship.

A. Emotion categories. To model facial expression signals of the six classic emotion categories, we computed MI between each Action Unit (either present or absent on each trial) and each of the participant's emotion category perceptual responses, represented as a binary coding (i.e., "happy" versus "not happy"). A high MI value indicates that the AU is strongly associated with (i.e., predicts) the participant's emotion category perceptual response; a low MI value indicates a weak association. We pooled trials across intensity ratings to derive a facial expression model that is not specific to intensity. To determine statistical significance, we used a non-parametric permutation test and the method of maximum statistics to correct for multiple comparisons.[107] Specifically, we randomly shuffled the participant's perceptual responses, re-calculated the MI value for each AU, and took the maximum MI value across all 42 AUs. We repeated this procedure for 1,000 iterations to derive a distribution of maximum MI under the null hypothesis that the presence of the AU is independent of the participant's perceptual responses. We rejected this hypothesis for AUs with MI values above the 95th percentile (Family-Wise Error Rate [FWER] over 42 AUs, $p < 0.05$, one-tailed). We applied this procedure to the data of each participant, resulting in a total of 360 facial expression models (60 participants x 6 emotion categories). Each facial expression model is represented as a 1 × 42-dimensional binary vector that details the AUs significantly associated with the participant's emotion category perceptual responses. Specifically, each vector element represents one of the 42 individual AUs and is coded as 1 or a 0 according to whether the AU is statistically significantly associated with the participant's emotion category perceptual response—e.g., "happy." For example, a facial expression model of "happy" that is composed of lip corner puller (AU12), cheek raiser (AU6), and brow raiser (AU1-2) would be represented as a binary vector by coding the vector elements associated with these AUs as 1 and all other elements as 0, thus producing a specific pattern of 1 and 0-coded vector elements. Representing each facial expression model—i.e., each AU pattern—in a common vector space thus enables objective comparisons. Figure S1A shows the results as color-coded matrices and corresponding face maps below.
B. Dimensions of valence and arousal. We used a similar procedure to model the facial expression signals that elicit the perception of the dimensions of valence and arousal. First, we measured the MI between each AU and the participant's dimensional rating responses for valence and arousal separately, and determined statistical significance using a non-parametric permutation test and the method of maximum statistics to correct for multiple comparisons[107] (Family-Wise Error Rate [FWER] over 42 AUs, $p < 0.05$, one-tailed). Next, for each AU with a significantly high MI value, we measured the point-wise mutual information (PMI) between the AU and each level of rating response—for example, "high arousal"—to reveal the specific AU-response relationship that underlies the overall MI value. A positive PMI value indicates that the presence of an AU (e.g., upper lid raiser, AU5) increases the probability of observing a specific response (e.g., "high arousal"). A negative PMI value indicates that the AU (e.g., lip corner puller-cheek raiser, AU12-6) decreases the probability of observing a specific perceptual response (e.g., "very negative" valence). To ensure enough trials for each rating level, we first re-scaled each participant's responses from 7 to 5 bins by iteratively combining the smallest neighboring ratings. After computing PMI, we established statistical significance using the same non-parametric permutation test described above and a two-tailed test to identify AUs that are associated, positively (above 97.5th percentile) or negatively (below 2.5th percentile; $p < 0.05$, two-tailed), with each level of valence and arousal for each participant separately. This resulted in a total of 400 facial expression models each for valence and arousal (40 participants x 5 levels of rating x 2 positive/negative associations; see Figure S1A, center and right panels, for results summed across participants). Finally, we built a 2-dimensional valence-arousal space of facial expression signals by building a facial expression for each of the 25 (i.e., 5 × 5) valence-arousal level combinations. For each of the 25 valence-arousal level combinations, we cross-combined in a pairwise manner all positively associated AUs across the two dimensions and removed any negatively associated AUs (see Figure S1B for an illustration). Notably, while most AUs elicit the perception of either valence or arousal, some AUs elicit the perception of valence *and* arousal—for example, nose wrinkler (AU9) is positively associated with "high arousal" and "negative valence" perceptual responses. We therefore restricted these AUs to these specific cells—e.g., nose wrinkler (AU9) only appears in "high arousal, negative valence" cells but never in "high arousal, positive" cells. We applied this procedure to the data collected from each individual participant, resulting in 40 such 5 × 5 valence-arousal facial expression signal spaces, thus resulting in a total of 1000 facial expression models (40 participants x 25 valence-arousal combinations). Each facial expression model is represented as a 1 × 42-dimensional binary vector that details the AUs significantly associated with the participant's dimensional responses. Figures 2B and S1B show the results displayed as face maps with results summed across 40 participants and normalized per face map for visualization purposes.

Facial expression mapping procedure

To examine whether facial expression signals of the six classic emotion categories are embedded into the valence-arousal dimensional space, we computed the pairwise correlation between facial expression signals of the emotion category (e.g., "happy") of each participant and facial expression signals of each valence-arousal dimensions of each participant. Specifically, we obtained six facial expression models—i.e., one for each emotion category—for each participant in the emotion categorization task, and 25 facial expression models—i.e., one per 5 valence levels x 5 arousal levels—for each participant in dimensional rating task (see Facial expression modeling procedure—STAR Methods). Each facial expression model is represented as a 42-dimensional binary vector as described above. We then computed the correlation between each facial expression model from the emotion categorization task (6 emotions x 60 participants = 360 in total) and each facial expression model from the dimensional rating task (5 valence levels x 5 arousal levels x 40 participants = 1000 in total). The Pearson correlations between each pair of binary vectors thus measures the similarity of their AU patterns. This generated 2400 correlation values for each of the six emotions in each cell of the valence-arousal

space (60 facial expression models per emotion category x 40 facial expression models per dimensional message). We then averaged these 2400 values to obtain an overall similarity measure between the facial expression models of each emotion category and each cell of valence and arousal space.

To test the generalization of the facial expression signal mapping results with the six classic emotion categories, we mapped a broader set of facial expression models of 19 complex emotion categories[3] onto the facial expression models of valence-arousal dimensions. Specifically, we measured the average similarity (i.e., Pearson correlation) between all facial animations categorized as a given complex emotion (e.g., "delighted") in the experiment (minimum 370 trials, maximum 6383 trials, median 1975 trials per emotion label across all complex emotions) and each of the 40 facial expression models in each cell of the valence-arousal dimensional space. This produced a pattern of correlation values distributed across the valence-arousal space for each complex emotion category (see Figure S1C for results). The systematic mapping observed validates the embedding of the facial expression signals of emotion categories into those of dimensions.

Validation of facial expression mappings

To validate the facial expression signal mappings, we compared the correlation pattern derived for each emotion category to the location of the corresponding emotion word. First, we extracted the average (mean) and standard deviation valence and arousal rating of each emotion word from an existing word corpus[44] obtained from English speaking participants using a 9-point rating scale. We then projected the 9-point ratings onto a 5-point scale to equate it with the valence-arousal space. Next, we plotted each emotion word into the valence-arousal space using the average and standard deviation values. Figure 2C shows the results as black crosses. We then compared the location of the emotion word in the 2-dimensional space with the location of the maximal correlation value of the facial expression signal (represented in Figure 2C as high saturation red)—each represented according to their distance to the origin point (valence = 0 and arousal = 0)—by fitting a linear regression between these values for all six emotion categories. Results showed a statistically significant association between the facial expression signal mappings and the semantic location of the correspond emotion words ($p = 0.0212$, two-tailed). Figure 2D shows the results in red, which confirms that the mapping of the facial expression signals of emotion categories onto those of dimensions corresponds with the semantic mapping of the emotion category word into the valence-arousal space. For example, the emotion category word "happy" is rated as "positively valenced" with "moderately high arousal" and thus located in the center right of the valence-arousal space (in Figure 2C, "happy" subplot). Similarly, the facial expression signals of "happy" correlate most strongly with those of "positive valence" and "moderately high arousal" (in Figure 2C, "happy" subplot, see red squares on center right). To test the generalizability of these results, we applied the same analysis to the facial expression signals of 19 more complex emotions and found similar results ($p = 0.00418$, two-tailed; see Figure 2D for results, shown in blue).

Experiment II

Conditional Mutual Information analysis

To identify the individual facial movements that specifically elicit the perception of emotion (1) categories, (2) dimensions, or (3) categories and dimensions, we used conditional mutual information (CMI), which measures the statistical relationship between two independent variables while controlling for the effects of a third variable. For example, in measuring the relationship between a given AU (e.g., lip corner puller, AU12) and the participants' emotion category perceptual responses, CMI measures this relationship while controlling for the influence of the participants' dimensional perceptual responses, henceforth represented as $CMI(AU12; Emotion\ Categories \mid Emotion\ Dimensions)$. Therefore, CMI can segregate out and thus precisely characterize the perceptions elicited in the receiver. To do so, we computed two CMI quantities: (1) CMI between the AU and the participants' emotion category responses while controlling for the effects of their dimension responses, represented as $CMI(AUX; Emotion\ Categories \mid Emotion\ Dimensions)$, and (2) vice versa, henceforth represented as $CMI(AUX; Emotion\ Dimensions \mid Emotion\ Categories)$. For example, to characterize the relationship between lip corner puller (AU12) and the participants' responses, we first compute $CMI(AU12; Emotion\ Categories \mid Emotion\ Dimensions)$. Specifically, we computed the MI between each AU and the participants' emotion category perceptual responses under each of the twenty-five dimensional response events, thus producing 25 sub-CMI values per emotion category. We then computed CMI as the weighted sum of the 25 sub-CMI values according to the probability of each response. A high CMI value indicates a statistical relationship between AU12 and the participants' emotion category perceptual responses even when their dimensional responses are known (i.e., fixed, and thus controlled). We can thus infer that AU12 provides information about the participants' emotion category perceptual responses in addition to any information it provides about their dimensional responses. In contrast, a low CMI value indicates that there is no such statistical relationship—i.e., we can thus infer that AU12 does not provide information about the participants' emotion category perceptual responses in addition to any it provides about their dimensional responses. Next, we compute CMI in the other direction: $CMI(AU12; Emotion\ Dimensions \mid Emotion\ Categories)$ using the same procedure. As before, a high CMI value indicates a statistical relationship between AU12 and the participants' dimensional responses when their emotion category perceptual responses are known. We can thus infer that AU12 provides information about the participants' dimensional responses in addition to any information it provides about their emotion category perceptual responses. In contrast, a low CMI value indicates that there is no such relationship—i.e., we can thus infer that AU12 does not provide information about the participants' dimensions responses in addition to any information it provides about their emotion category perceptual responses. We determined the statistical significance of all resulting CMI values using a non-parametric permutation test and the method of maximum statistics to correct for multiple comparisons as described above. Therefore, by computing CMI in both directions we obtained two CMI values for each AU corresponding to direction (1) and direction (2) that characterizes each AU in one of four ways:

AUs with (1) high CMI in the first direction and low CMI in the second direction, referred to as 'category signals;' (2) low CMI in the first direction and high CMI in the second direction, referred to as 'dimensional signals;' (3) high CMI in both directions, referred to as 'multiplex signals,' which can elicit the perception of both emotion categories and dimensions; and (4) low CMI in both directions, which indicates that the AU does not provide information about the receivers' emotion category or dimensional responses. Figure 3A shows the three sub-sets of AUs that are statistically associated with participants' responses, displayed as a color-coded matrix (see also Figure S3A). Figure S3B shows results confirming the AUs that do not systematically elicit the perception of emotion categories or dimensions. Re-computation of CMI according to the sex of the stimulus face and the sex of the participants showed results that are consistent with the group-level sex-pooled results, whereby the majority of Action Units comprised multiplexed signals as defined above. We further specified the emotion categories and/or dimensional messages that each AU elicits the perception of using point-wise mutual information (PMI),[12] which quantifies the contribution of each possible event—e.g., the six emotion categories—to the overall CMI value computed above. Figure 3A shows the results as color-coded face maps; Figure S3A, center and right panels, show a detailed breakdown.

Perceptual link—emotion categories and dimensions

Before conducting the CMI analysis as described above, we first tested for the robust finding that emotion category responses to facial expressions correlate with (i.e., predict) dimensional responses to the same facial expressions—for example, facial expressions categorized as "anger" are often also rated as "negative valence, high arousal."[6–8] To ensure enough trials for each level of the valence and arousal ratings, we re-binned each participant's ratings from 7 to 5 bins for valence and arousal separately by iteratively combining the lowest occupancy bin with its lowest occupancy neighbor. Next, we represented each participant's joint valence and arousal ratings as a single, combined variable comprising 25 unique events—for example, where a participant rated a facial expression as 4 for arousal and 5 for valence, the trial would be represented as a single value (e.g., '24'). We then computed, for each of 20 individual participants, the statistical dependence between their emotion category perceptual responses (six possible emotion categories) and their dimensional responses (25 possible arousal and valence events) using mutual information (MI) and established statistical significance using a non-parametric permutation test as described above. Specifically, we randomly shuffled the participant's dimensional responses before re-calculating MI for each participant and repeated this procedure for 1,000 iterations per participant. This produced a distribution of MI values under the null hypothesis that the participant's emotion category perceptual responses are independent from their dimensional responses. We rejected this hypothesis for participants with MI values above the 95th percentile of the randomly generated MI distributions ($p < 0.05$). Results showed, for each of 20 participants, a statistically significant relationship between the participant's emotion category responses and their dimensional responses, suggesting a close link between these perceptions. Figure S2A shows the results.

We further characterized the relationship between the participant's emotion category and dimensional responses by specifying the range of dimensional ratings associated with each emotion category using PMI as described above. We consider PMI values for the presence of the considered emotion response, represented as one versus the rest binary coding per emotion (e.g., "happy" versus "not happy"). A high PMI value indicates that the perception of a given emotion category such as "happy" is associated with a given set of dimensional responses such as "positive valence, high arousal;" a low value indicates that they are not related—for example, perceptions of the emotion category "anger" is dis-associated with dimensional responses such as "positive valence, high arousal." We computed the PMI between each of the six emotion category responses and each of the 25 valence-arousal events for each participant separately. Figure S2A center panel shows the results for each emotion category, averaged across participants. Results characterized each emotion by specific location in the valence and arousal space. For example, facial movements categorized as "happy" are also primarily rated as positively valenced, ranging from low to high arousal and rarely rated as negative valence. In contrast, facial movements categorized as "disgust" are primarily rated as negatively valenced, ranging from low to high arousal and rarely perceived as positively valenced. A visual inspection of these distribution patterns suggests that the perception of each emotion category is associated with a specific range of valence and arousal ratings, thus forming distinct patterns. To test this formally, we measured the pairwise similarities between the patterns of positive relationships of each of the six emotion categories using standard Euclidean distance. Figure S2B shows the results. As shown by these similarity values, each emotion category response is associated with a distinct pattern of dimensional responses with overlap between "disgust" and "anger," as is commonly reported.[13,108–110] In sum, analysis of the participants' perceptual responses shows that facial expressions perceived as an emotion category such as "happy," "anger," or "sad" are also systematically perceived according to a specific range of dimensions such as "low to high arousal, positive valence," "low to high arousal, negative valence," or "low arousal, negative valence," respectively. These similarity distribution patterns also closely mirror the patterns of facial movements of emotion categories embedded into the valence-arousal facial movement space (see Figure 2B). Together, these results demonstrate the close relationship between the perception of emotion categories and dimensions, thereby mirroring existing work.[51–53]

Current Biology, Volume *32*

Supplemental Information

Facial expressions elicit multiplexed perceptions of emotion categories and dimensions

Meng Liu, Yaocong Duan, Robin A.A. Ince, Chaona Chen, Oliver G.B. Garrod, Philippe G. Schyns, and Rachael E. Jack

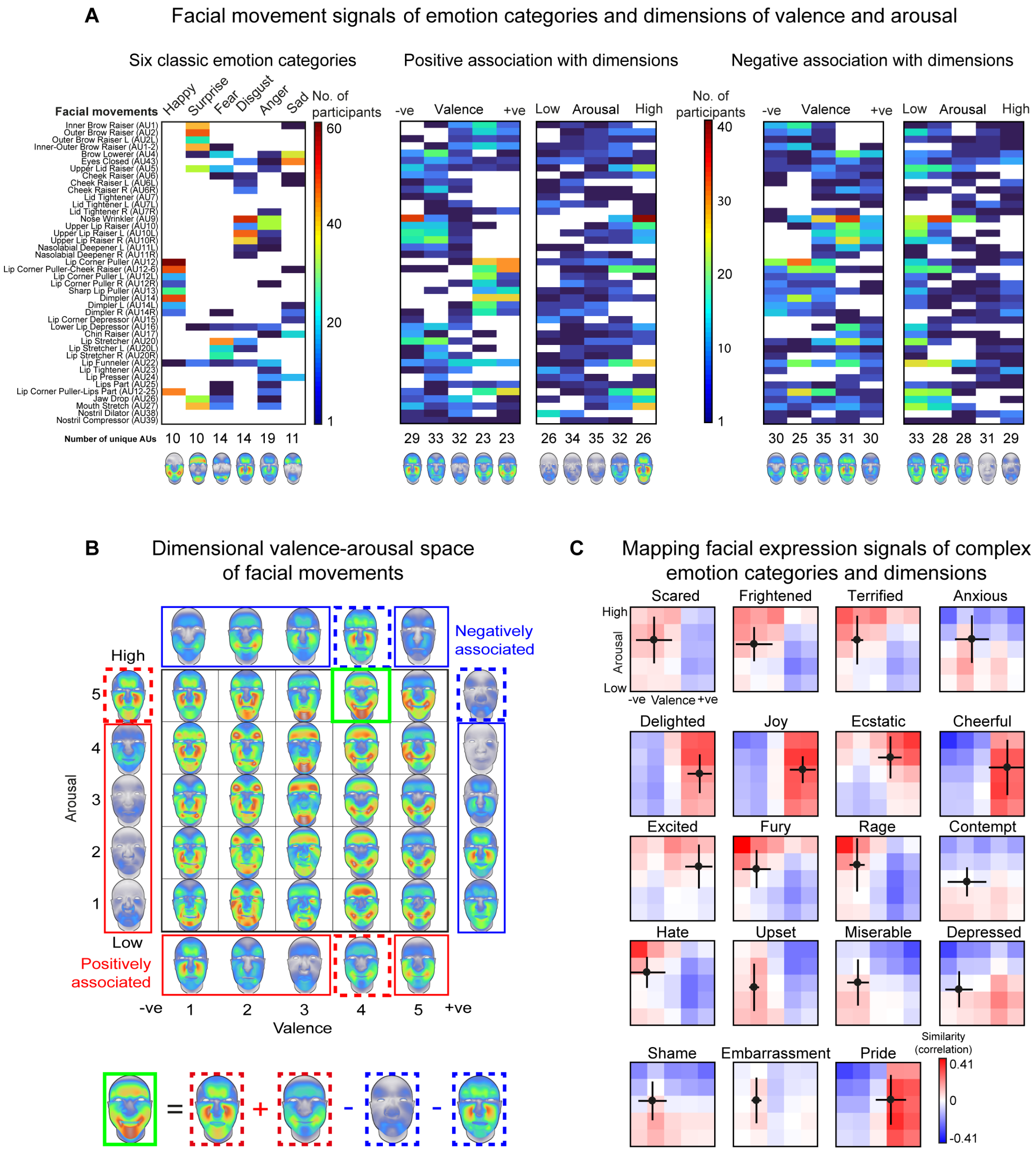

Figure S1. Modelling facial expression signals of emotion categories, and of emotion dimensions plus their mapping, Related to Figure 2, Experiment I in STAR Methods. (A) Facial movement signals of emotion categories and dimensional information. Each color-coded matrix shows the individual facial movements (i.e., Action Units) that signal emotion categories (left) or dimensional information (centre, right) as determined using Mutual Information (MI)[S1]. Color-coding refers to the number of participants (see colorbars). Warmer colors indicate higher numbers, cooler colors indicate lower numbers – for example, in emotion categories, Lip Corner Puller (AU12) is associated with 'happy' in all 60 participants (represented in dark red). For dimensional information, each matrix shows the AUs that are positively associated with perceptions of valence and arousal (centre) and negatively associated (right). For example, Nose Wrinkler (AU9) is positively associated with negative valence and negatively associated (i.e., disassociated) with positive valence and/or low arousal amongst most participants (represented in dark red – see color bar in centre). The total number of unique AUs and corresponding face

maps are shown below each emotion category and each dimensional rating. (B) Dimensional valence-arousal space of facial movements. Face maps in each cell of the 5 x 5 matrix show the facial movements associated with each combined level of valence and arousal, summed across participants. Each facial expression (see example outlined in green) is a combination of the corresponding positively associated individual Action Units (see dashed red outline) with any negatively associated AUs (dashed blue outline) removed. An example is shown below. (C) Mapping facial expression signals of complex emotions onto dimensions. Each color-coded sub-plot shows the average correlation between the facial expressions of a complex emotion (e.g., ‘ecstatic’) and the facial expressions of the dimensional valence-arousal space. Red indicates positive correlations; blue indicates negative correlations (see color bar on bottom right). For example, the facial expression of ‘ecstatic’ is positively correlated with facial expressions of high arousal positive valence, and negatively correlated with low arousal negative facial expressions. The semantic location of each emotion word is shown as a black point with crosses representing the standard deviation for each dimension[S2].

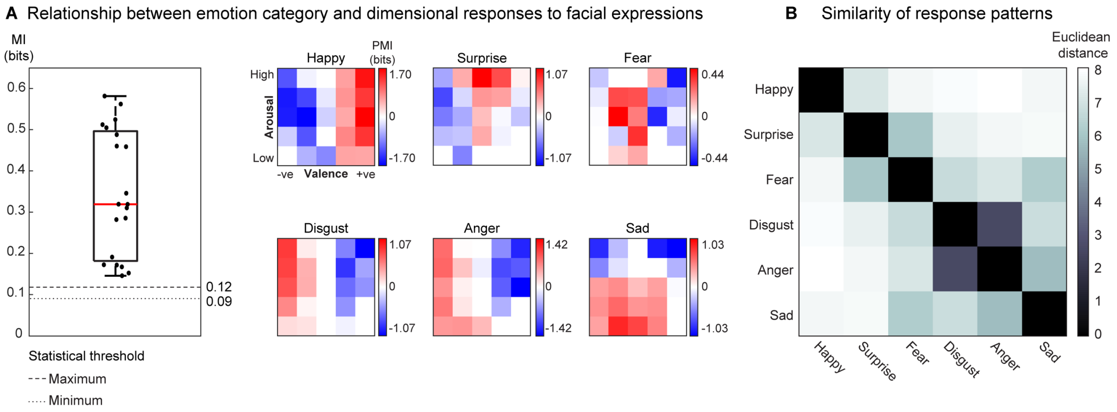

Figure S2. Relationship between emotion category responses and dimensional responses to facial expressions, Related to Experiment II, Replicating the link between the perception of emotion categories and dimensions in STAR Methods. (A) Relationship between emotion category and dimensional responses to facial expressions. The boxplot shows the Mutual Information (MI)[S1] values computed between the participants’ emotion category responses and their dimensional responses to the same facial expressions. Black points represent individual participants; the red line indicates the median; dashed lines show the minimum and maximum thresholds for statistical significance across participants. Color-coded matrices to the right show the specific relationship between the participants’ emotion category responses and their dimensional responses, measured using Pointwise Mutual Information (PMI)[S3]. Red indicates positive relationships; blue indicates negative relationships (see color bar to right) – for example, facial expressions categorized as ‘happy’ are often rated as ‘positive valence’ with varying levels of arousal. (B) Similarity of response patterns. To formally test the distinctiveness of these category-dimensional response relationships, we measured the pairwise similarities between the six distribution patterns. Results are shown as a gray-scale matrix. Darker squares indicate higher similarities; lighter squares indicate lower similarities (see colorbar to right). Results confirm that each emotion category is associated with a distinct range of valence and arousal ratings with overlap between disgust and anger, as commonly reported[S4–6].

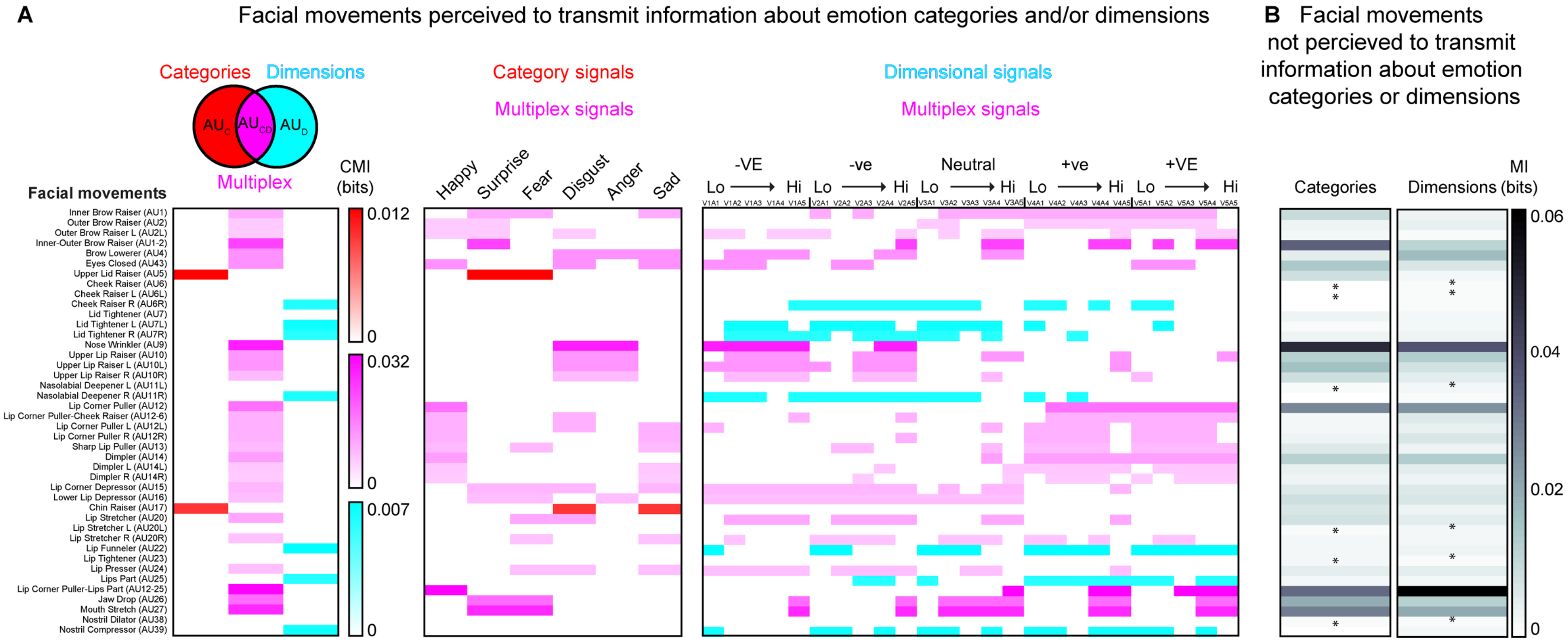

Figure S3. Facial movement signals that transmit information about emotion categories and/or dimensions, Related to Figure 3, Experiment II, Conditional Mutual Information analysis in STAR Methods. (A) Facial movement signals of emotion category and dimensional information. Left panel – each AU (see labels on y axis) is color-coded according to the categorical and/or dimensional information it transmits (see Venn diagram), derived using Conditional Mutual Information. Color saturation represents the CMI value, averaged across participants (see color bars to right). Centre and right panels – each color-coded matrix shows the specific emotion categories (represented in red, magenta) and dimensional information (represented in cyan, magenta) transmitted by each AU, derived using Pointwise Mutual Information (PMI). For example, Upper Lid Raiser (AU5) is an emotion category signal (left panel, red) that transmits information about the emotion categories 'fear' and 'surprise' (centre panel, red); the unilateral Lid Tighteners (AU7L/R) are dimensional signals (left panel, cyan) that transmit information about 'negative valence' across varying levels of arousal (right panel, cyan); Nose Wrinkler (AU9) is a multiplex signal (left panel, magenta) that transmits information about the emotion categories 'disgust' and 'anger' (centre panel, magenta) and the dimensions of 'negative valence' across varying levels of arousal (right panel, magenta). (B) Facial movements that do not transmit information about emotion categories or dimensions. Action Units identified by the CMI analysis as not influencing participant responses are indicated by asterisks. Gray-scale coding shows the Mutual Information (MI) between each AU and the participants' responses for the emotion categorization task (left column) and dimension rating task

(right column; see color bar on right). All AUs marked with an asterisk have low MI values in both tasks, confirming that they do not influence the participants’ responses.

SUPPLEMENTAL REFERENCES

S1. Ince, R.A.A., Giordano, B.L., Kayser, C., Rousselet, G.A., Gross, J., and Schyns, P.G. (2017). A statistical framework for neuroimaging data analysis based on mutual information estimated via a gaussian copula. Hum. Brain Mapp. *38*, 1541–1573.

S2. Warriner, A.B., Kuperman, V., and Brysbaert, M. (2013). Norms of valence, arousal, and dominance for 13,915 English lemmas. Behav. Res. Methods *45*, 1191–1207.

S3. Bouma, G. (2009). Normalized (pointwise) mutual information in collocation extraction. Proc. GSCL, 31–40.

S4. Jack, R.E., Garrod, O.G.B., and Schyns, P.G. (2014). Dynamic Facial Expressions of Emotion Transmit an Evolving Hierarchy of Signals over Time. Curr. Biol. *24*, 187–192.

S5. Aviezer, H., Hassin, R.R., Ryan, J., Grady, C., Susskind, J., Anderson, A., Moscovitch, M., and Bentin, S. (2008). Angry, disgusted, or afraid? Studies on the malleability of emotion perception. Psychol. Sci. *19*, 724–732.

S6. Widen, S.C., Russell, J.A., and Brooks, A. (2004). Anger and disgust: Discrete or overlapping categories. In 2004 APS Annual Convention, Boston College, Chicago, IL.
